# Investigating the role of the *Listeria monocytogenes* noncoding RNA Rli47 during the response to environmental stressors

**DOI:** 10.1093/femsmc/xtaf012

**Published:** 2025-10-20

**Authors:** Bienvenido W Tibbs-Cortes, Jessica L Strathman-Runyan, Stephan Schmitz-Esser

**Affiliations:** Infectious Bacterial Diseases Unit, Agricultural Research Service, USDA, Ames, IA, 50011, United States; Interdepartmental Microbiology Graduate Program, Iowa State University, Ames, IA, 50011, United States; Department of Animal Science, Iowa State University, Ames, IA, 50011, United States; Interdepartmental Microbiology Graduate Program, Iowa State University, Ames, IA, 50011, United States; Department of Animal Science, Iowa State University, Ames, IA, 50011, United States; Interdepartmental Microbiology Graduate Program, Iowa State University, Ames, IA, 50011, United States; Department of Animal Science, Iowa State University, Ames, IA, 50011, United States

**Keywords:** *Listeria monocytogenes*, Rli47, transcriptome, stress response, noncoding RNA, food safety

## Abstract

*Listeria monocytogenes* is a food-borne pathogen that can cause severe disease in immunocompromised persons, and its ability to survive stressors encountered in food production environments (FPEs) makes it difficult to eliminate from the food chain. Previous transcriptomic analysis revealed that in response to lactic acid exposure, *L. monocytogenes* significantly upregulates Rli47, a noncoding RNA that has previously been shown to interact with the *ilvA* transcript and suppress growth of *L. monocytogenes* in the absence of isoleucine. We show that at logarithmic phase, an *rli47* deletion mutant had a higher survival compared to the parent strain after exposure to lactic acid. Flow cytometry indicated that lactic acid exposure did not differentially affect the proportion of metabolically active cells in the deletion mutant and wild type. Transcriptomic analysis and *in silico* target prediction suggested that Rli47 could affect pathways involved with cell envelope structure; due to the link between cell envelope integrity and organic acid stress, it is possible that in the absence of *rli47* the cell envelope of logarithmic phase *L. monocytogenes* cells is more resistant to lactic acid exposure. These results suggest that Rli47 functionality may vary due to factors including temperature and nutrient availability.

## Teaser

The noncoding RNA Rli47 is involved in the *Listeria monocytogenes* stress response.

## Author summary

*Listeria monocytogenes* is a bacterial pathogen that can cause serious and even fatal disease in the immunocompromised. Despite measures taken by food producers to mitigate bacterial contamination, *L. monocytogenes* is capable of surviving in food production environments, (FPEs) contaminating food products, and causing outbreaks. Genetic analysis and molecular characterization of *L. monocytogenes* seek to better understand how this pathogen is capable of resisting sanitation measures in order to improve food safety. In this study, we investigate Rli47, a noncoding RNA that is known to regulate amino acid biosynthesis *L. monocytogenes*, and how it relates to stress survival. We observed that bacteria without *rli47* survive the food preservative lactic acid at a higher rate than bacteria with *rli47*. Further research suggested that this could be due to interactions between Rli47 and the *L. monocytogenes* cell envelope. Our work identifies a role for Rli47 in stress response and suggests that the function of this noncoding RNA may be affected by environmental conditions.

## Introduction

*Listeria monocytogenes* is a Gram-positive pathogen that causes listeriosis, a disease characterized by systemic infection resulting in severe symptoms including sepsis, meningitis, and encephalitis. The immunocompromised and elderly are particularly vulnerable (Schlech 2019). Infection by *L. monocytogenes* occurs through consumption of contaminated foods, and preventing *L. monocytogenes* contamination is therefore a high priority in FPEs. However, *L. monocytogenes* possesses an array of genetic mechanisms that allows it to survive and grow despite many antimicrobial measures. Studying the *L. monocytogenes* stress response and its genetic mechanisms may therefore provide new avenues for preventing *L. monocytogenes* outbreaks (Milillo et al. 2012, NicAogáin and O’Byrne 2016).

The small noncoding RNA (sRNA) Rli47 was originally identified and characterized in *L. monocytogenes* strain EGD-e, where it was upregulated in cultured stationary phase cells and in cells recovered from the murine intestine. Subsequent research demonstrated that *rli47* was also at least partially dependent on σ^B^, the alternative sigma factor important for *L. monocytogenes* virulence and stress response (Oliver et al. 2009, Toledo-Arana et al. 2009). Later studies also observed upregulation of *rli47* during mid-logarithmic phase (Mujahid et al. 2013), in *L. monocytogenes* in macrophages (Mraheil et al. 2011), in an *L. monocytogenes* mutant not expressing the soil-associated quorum-sensing molecule AgrA (Vivant et al. 2015), during cocultivation of *L. monocytogenes* with *Lacticaseibacillus casei* in gnotobiotic mice (Archambaud et al. 2012), and during cocultivation with a *Psychrobacter* strain on Brain Heart Infusion (BHI) agar plates (Anast and Schmitz-Esser 2020). Homologs of *rli47* have since been found across *L. monocytogenes* strains and in the species belonging to the *sensu stricto* group of the genus *Listeria* (Oliver et al. 2009, Marinho et al. 2019).

Because it is part of the σ^B^ regulon, the first functional analysis of Rli47 focused on its role in several stress conditions, but no significant difference in phenotype was observed between an Δ*rli47* mutant and its parent strain in response to salt, cold, oxidative, or inorganic acid stresses. Although transcriptomics and proteomics indicated that Rli47 regulated an uncharacterized operon containing *lmo0636* and *lmo0637*, subsequent experiments could not demonstrate interaction between Rli47 and the *lmo0636* transcript (Mujahid et al. 2013). More recent functional analysis revealed that Rli47 binds to the Shine–Dalgarno region of the transcript of *ilvA*, a gene encoding a threonine deaminase that catalyses the first step in the isoleucine biosynthesis pathway. An *rli47* deletion mutant possessed a shorter lag phase compared to the wild type when grown in minimal media without isoleucine, suggesting that Rli47 suppresses *L. monocytogenes* growth under conditions where isoleucine is limited, perhaps as a means of controlling growth under adverse conditions. Intriguingly, the *rli47* deletion mutant also differentially expressed additional metabolic pathway genes, suggesting that Rli47 has additional roles (Marinho et al. 2019).

The transcriptomics and growth rate experiments performed by Marinho et al. (2019) were conducted at 37°C in a chemically defined medium, and it is therefore unknown if Rli47 acts similarly under conditions encountered by *L. monocytogenes* in FPEs (e.g. lower temperatures, pH extremes, more complex nutrient profiles, and competition with other organisms). Previous transcriptome analyses indicate that Rli47 may be involved in the response to FPE-relevant stressors. We previously observed that *rli47* was highly upregulated in response to lactic acid stress in *L. monocytogenes* strains 6179 and R479a (Cortes et al. 2020). A later study utilizing the strains RO15 and ScottA similarly observed large proportions of reads in the transcriptome mapping to *rli47* following high-pressure processing (Duru et al. 2021). A similar phenomenon of high *rli47* transcript abundance was observed for *L. monocytogenes* cocultivated for 72 hours alongside a *Psychrobacter* strain; *rli47* was also significantly more expressed in cocultivation than in monoculture (Anast and Schmitz-Esser 2020).

To investigate the potential role of *rli47* in FPE-relevant stress, we compared wild-type *L. monocytogenes* 6179 to its isogenic Δ*rli47* mutant grown to either logarithmic or stationary phase; strains were subsequently exposed to 20°C, lactic acid, and hydrogen peroxide (H_2_O_2_). These stress conditions were chosen based on their relevance in FPEs. Organic acids including lactic acid and its salts such as sodium lactate are often used as food additives in the food industry to inhibit the growth of *L. monocytogenes* (Bucur et al. 2018). During cleaning and sanitation procedures in FPEs, *L. monocytogenes* is also frequently exposed to oxidizing agents. Organic acids in the small intestine and oxidative stressors in macrophages are also encountered by *L. monocytogenes* during its pathogenic lifecycle (Ferreira et al. 2003, Mains et al. 2021).

6179Δ*rli47* exhibited higher survival than the wild type following exposure of logarithmic phase cells to lactic acid. However, no differences were observed between the proportion of metabolically active 6179 and 6179Δ*rli47* cells following lactic acid exposure as measured by flow cytometry, and transcriptomic analysis indicated few differences between the two strains under multiple conditions. Transcriptomics and *in silico* modeling suggest that Rli47 may have indirect effects on cell wall stability and therefore lactic acid survival. Together, these analyses add to our understanding of Rli47 in the stress response and general physiology of *L. monocytogenes*.

## Methods

### Bacterial strains and maintenance

A list of all strains used in this work can be found in Table 1. *Listeria monocytogenes* 6179 belongs to serotype 1/2a, sequence type 121, and was originally isolated from a cheese production plant in Ireland. Due to being repeatedly isolated from this facility over multiple years, it is considered a persistent isolate (Fox et al. 2011, Tibbs-Cortes et al. 2023). Numerous studies of 6179 have been conducted which characterize its genetics and its resistance to multiple antimicrobials used in food production (Fox et al. 2011, Müller et al. 2013, Casey et al. 2014, Schmitz-Esser et al. 2015, Harter et al. 2017, Naditz et al. 2019, Cortes et al. 2020, Tibbs-Cortes et al. 2023). *Listeria monocytogenes* 6179 and 6179Δ*rli47* were maintained at 20°C on tryptic soy agar (TSA) or in tryptic soy broth (TSB) unless otherwise stated. *Escherichia coli* StrataClone Solopack Competent Cells (Agilent) were used for molecular cloning and were cultivated at 37°C in lysogeny broth (LB) or on LB plates. All broth cultures were incubated with 200 rpm shaking. The plasmid pHOSS1 and constructs with the pHOSS1 backbone were maintained in media with 150 μg/ml ampicillin for *E. coli* or 15 μg/ml erythromycin for *L. monocytogenes*. For pNZ44 and derivative constructs, chloramphenicol was used at concentrations of 25 μg/ml for *E. coli*, 10 μg/ml for *L. monocytogenes* in broth, and 15 μg/ml for *L. monocytogenes* on plates.

**Table 1.** Strains and plasmids used in this study.

Strain	Details
*L. monocytogenes* 6179	ST121 isolate from an Irish cheese plant (Fox et al. 2011)
*L. monocytogenes* 6179Δ*rli47*	6179 with a deletion of *rli47* from the transcription start site to the beginning of the 3′ Rho-dependent terminator
*L. monocytogenes* 6comprli	6179Δ*rli47* harboring p44rli
*E. coli* Strataclone	*E. coli* strain used for cloning and maintenance
**Plasmid**	**Details**
pHOSS1	Allelic-exchange mediating vector for *L. monocytogenes*. Thermosensitive *ori* with anti-*secY* marker for counterselection (Abdelhamed et al. 2015)
pHOSS1rli	pHOSS1 harboring a mutant allele with the *rli47* deletion
pNZ44	Constitutive expression vector for *L. monocytogenes* (de Ruyter et al. 1996, McGrath et al. 2001)
p44rli	pNZ44 harboring *rli47* for complementation

### General PCR methods

All primers used in this study are described in Table S1, and annealing temperatures for each primer pair are listed in Table S2. For molecular cloning, Polymerase Chain Reactions (PCRs) were conducted using the Phusion High-Fidelity DNA Polymerase (Thermo Scientific). Platinum II Taq DNA Polymerase (Thermo Scientific) was used for diagnostic PCRs. PCR reaction mixtures and cycling conditions are described in Tables S3 and S4, respectively.

### Generation of an *rli47* deletion mutant in *L. monocytogenes* 6179

#### Splicing by overlap extension PCR

The pHOSS1 suicide vector was utilized to perform allelic exchange-mediated deletion of *rli47* from the *L. monocytogenes* 6179 chromosome (Abdelhamed et al. 2015). To facilitate pHOSS1 integration into the chromosome for subsequent allelic exchange, splicing by overlap extension PCR was used to create a ∼2 kb fragment with a deletion of bases 1–485 of *rli47* [relative to the TSS predicted by Marinho et al. (2019)] but otherwise homologous to the *rli47* locus. This deletion removed the entire predicted *rli47* transcript except for the rho-dependent terminator shared with *lmo2142*, similar to the mutant generated by Marinho et al. (2019). PCRs were performed to amplify ∼1 kb fragments flanking *rli47* from *L. monocytogenes* 6179 genomic DNA; amplifications with primer pairs RA/RB3 and RC2/RD yielded fragments AB3 and C2D, respectively. Amplifications were performed under the following conditions: (1) initial denaturation—98°C, 3 minutes; (2) denaturation—98°C, 30 seconds; (3) annealing (see Table S3 for annealing temperatures for primer pairs), 30 seconds; (4) elongation—72°C, 75 seconds; and (5) final elongation—72°C, 10 minutes. These PCR products were purified by gel electrophoresis and cleaned with a GeneJET Gel Extraction Kit (Thermo Scientific) according to the manufacturer’s instructions. The concentrations of the fragments were measured using a NanoDrop 2000 (Thermo Scientific).

RB3 and RC2 incorporated complementary 24 bp regions at the 3′ end of AB3 and the 5′ end of C2D to facilitate splicing. The purified fragments were then mixed in a 1:1 ratio and subsequently diluted 1:20 in PCR-grade H_2_O. Initial annealing and splicing of AB3 and CD2 was performed by mixing 1 μl of the diluted fragment mixture with Phusion High-Fidelity DNA Polymerase and all necessary reagents except for primers. This first stage of annealing and amplification was conducted using the following conditions: (1) initial denaturation—98°C, 30 seconds; (2) denaturation—98°C, 10 seconds; (3) annealing—55°C, 30 seconds; and (4) elongation—72°C, 80 seconds. Steps 2–4 were repeated for a total of 15 cycles. After 15 cycles, primers RA and RD were added to the mixture, and the final product was amplified under the following conditions: (1) initial denaturation—98°C, 30 seconds; (2) denaturation—98°C, 10 seconds; (3) annealing—67.3°C, 30 seconds; (4) elongation—72°C, 80 seconds; and (5) final elongation—72°C, 10 minutes. Steps 2–4 were repeated for a total of 35 cycles. The final ∼2 kb product lacking *rli47*, referred to as Rli47AD, was confirmed by gel electrophoresis and purified by gel purification as described above.

#### Construction of a pHOSS1 vector for allelic exchange

Rli47AD and the plasmid pHOSS1 were digested with NcoI and SalI (Thermo Scientific). For digested Rli47AD, restriction enzymes were inactivated by heat treatment, and digested pHOSS1 was purified via gel purification. T4 DNA ligase (Thermo Scientific) was used to ligate Rli47AD into pHOSS1 per the manufacturer’s instructions. The ligation mixture was then cloned into *E. coli* StrataClone Solopack Competent Cells (Agilent) via heat shock for verification and maintenance. For all subsequent verification steps, PCRs were performed using Platinum II Taq Hot-Start DNA Polymerase (Invitrogen) according to the protocols listed in Tables S3 and S4. Transformants were screened for the pHOSS1 + Rli47AD construct by colony PCR with primers pHOSS1MCSF and pHOSS1MCSR to identify transformants with the ∼2 kb insert in pHOSS1 (Table S2). Plasmids were then extracted from these colonies using a Purelink Quick Plasmid Miniprep Kit (Invitrogen) and submitted for Sanger sequencing using the primers Rli47SeqF and Rli47SeqR. The confirmed construct was named pHOSSrli.

#### Allelic exchange-mediated deletion of *rli47*

Electroporation was used to transform pHOSSrli into *L. monocytogenes* 6179 per the protocols outlined in Monk et al. (2008). Briefly, *L. monocytogenes* 6179 was made electrocompetent by growth to logarithmic phase, subsequent exposure to 10 μg/ml penicillin G, and three washes in a 1 mM HEPES and 0.5 M sucrose solution. Electroporation was performed by adding 50 μl electrocompetent *L. monocytogenes* and 2 μl pHOSSrli to a 1 mm cuvette and electroporating at 1 KV, 25 μF, and 400 Ω using a Gene Pulser XCell with PC module (Bio-Rad). Transformants were recovered on BHI with erythromycin for ∼3 days at 30°C to select for pHOSSrli transformants before confirming the presence of the plasmid by PCR with pHOSS1MCSF and pHOSS1MCSR. The presence of pHOSSrli was further confirmed by screening isolates via colony PCR with the Rli47A and Rli47D primers for the presence of two bands representing the pHOSS1rli and wild-type alleles. Taking advantage of the pHOSS1 thermosensitive origin of replication, 6179 + pHOSSrli transformants were then incubated on BHI agar with 15 μg/ml erythromycin at 42°C for 2 days to select for cells which harbored integrated pHOSSrli. Colonies that grew were isolated and passaged under the same conditions for a total of three passages to ensure integration.

To enable selection for excision and loss of the plasmid, pHOSS1 harbors an antisense *secY* cassette under the control of an anhydrotetracycline promoter, which prevents production of the essential SecY protein when expressed. Colonies of 6179 with integrated pHOSSrli were first incubated in 2 ml BHI broth at 30°C overnight, and 20 μl of this culture was then used as an inoculum into fresh BHI. Cells were passaged in this manner for a total of three overnight cultures. 2 ml of BHI was inoculated with 20 μl of the final overnight at 42°C for 8 hours to cure nonintegrated pHOSSrli. The culture was then plated on BHI plus 1.5 μg/ml anhydrotetracycline and incubated for 3 days at 30°C. Colonies were then replica plated to test their sensitivity to erythromycin. Erythromycin-sensitive colonies were further screened for the absence of pHOSS1 using primers pHOSS1_fwd and pHOSS1_rev. The deletion of *rli47* from the chromosome was confirmed using primers Rli47ChromF and Rli47ChromR. To ensure that the excision and curing step had not also cured the native 6179 plasmid, a PCR screening for the presence of the 6179 plasmid gene *clpL* was performed using primers ClpLF and ClpLR. No colonies were found that lacked *clpL*. Colonies which were cured of pHOSS1 and lacked *rli47* were sent for Sanger sequencing using primers Rli47SeqF and Rli47SeqR, confirming the generation of a mutant lacking *rli47* from bases 1–485. This mutant was designated 6179Δ*rli47*.

### Validation of the 6179Δ*rli47* mutant

Sanger sequencing also identified a single nucleotide deletion of the wild-type adenine residue at position −7 relative to the *rli47* TSS in 6179Δ*rli47*, likely reflecting scarring from the allelic exchange process. Whole genome sequencing and subsequent variant calling was also performed to further validate 6179Δ*rli47*. Briefly, the DNeasy Powerlyzer Powersoil Kit (Qiagen) was used to extract long fragment gDNA from a pelleted overnight culture of 6179Δ*rli47* according to the manufacturer’s instructions but with two modifications. First, prior to beginning the kit workflow, the pellet was resuspended in 1 ml 1X PBS to which 60 μl of 1X lysozyme (Thermo Scientific) was added. The pellet was then incubated for 30 minutes at 37°C. Second, a Bead Mill 24 homogenizer (Fisher Scientific) was utilized for the homogenization step (a single 1-minute cycle with an intensity of 1.45). Extracted gDNA was sent to Plasmidsaurus for long-read sequencing on the Oxford Nanopore platform (v14 library preparation chemistry; R10.4.1 flow cell).

Long reads from 6179Δ*rli47* were utilized for variant calling using the 6179 parent strain as the reference. An updated assembly of 6179 was generated via Trycycler v0.5.6 from the long reads used to create GCF_028768565.1, the most recent 6179 assembly (SRA accession SRR20168270) (Wick et al. 2021). Trycycler assembled two closed contigs representing the 6179 chromosome and plasmid, which were subsequently polished by Polypolish v0.6.1 and pypolca v0.4.0 using the short reads associated with GCF_028768565.1 (SRA accession SRR20168271) (Wick et al. 2021, Wick and Holt 2022). *6179Δrli47* long reads were aligned against the new 6179 assembly using minimap2 v2.28 (Li 2018), and the resultant BAM files were used for variant calling by Clair3 v1.2.0 with the following options and model: –no_phasing_for_fa, –haploid_precise, –enable_variant_calling_at_sequence_head_and_tail, and r1041_e82_400bps_sup_v430_bacteria_finetuned (Zheng et al. 2022). Clair3 identified a single Single Nucleotide Polymorphism (SNP) possessing a variant call format (VCF) quality score over 20. This C>T substitution in 6179Δ*rli47* relative to the wild type was found at position 188 in *LM6179_00761* (*lmo0459*), an uncharacterized gene with a helix-turn-helix domain. While this SNP would result in a missense mutation of Ser63Leu in LM6179_00 761, this residue falls outside the predicted helix-turn-helix domain, suggesting it would not disrupt the domain. Furthermore, Ser63 was not predicted to be a site of phosphorylation based on analysis via MPSite (Hasan et al. 2019).To further determine if Ser63Leu impacted Lm6179_00761, we modeled the LM6179_00761 protein structure with and without Ser63Leu via the AlphaFold3 web server (https://alphafoldserver.com/) (Abramson et al. 2024). The crystallographic information files representing the two predicted structures were subsequently compared using the RCSB Pairwise Structure Alignment tool (https://www.rcsb.org/alignment) with the template modeling (TM)-align method (Bittrich et al. 2024). The resultant rrot mean square deviation (RMSD) and TM-score were 1.01 and 0.99, respectively, demonstrating that the topologies and structures of wild-type LM6179_00761 and Ser63Leu LM6179_00761 were extremely similar.

### Growth curve assays

Growth metrics of *L. monocytogenes* 6179 and 6179Δ*rli47* were determined at 37°C and 25°C (the latter temperature was used in place of 20°C due to the lack of a plate reader with cooling capabilities). For each experiment, three biological replicates of each strain were first grown to logarithmic phase at the corresponding temperature. 2 μl of cells were then used to inoculate 198 μl of TSB in a 96-well plate. This was repeated for a total of four technical replicates per biological replicate. Cells were then incubated in a BioTek Synergy H1 plate reader with 200 rpm shaking at the appropriate temperature. OD_600_ measurements were conducted every 30 minutes over 24 hours for the 37°C growth curve; for the 25°C experiment measurements were taken hourly over the course of 48 hours.

### Colony-forming unit assays for percent survival

The comparative survival of 6179 and 6179Δ*rli47* after exposure to lactic acid was determined using a colony-forming unit (CFU) assay. Strains were grown at 20°C in TSB to logarithmic phase (OD_600_ ∼0.25) to create a baseline culture, and 50 μl of cells baseline culture were then used to inoculate 4950 μl of TSB with lactic acid (final concentration 1% v/v) adjusted to pH 3.4–3.6. Cells were then exposed to stress for 2 hours at 20°C with 200 rpm shaking. The 2 hour time point was chosen because this duration of 1% lactic acid exposure was previously demonstrated to negatively affect 6179 (Naditz et al. 2019). Three samples of 100 μl were harvested from both the baseline culture and stressed culture, and these samples were serial diluted in phosphate buffered saline (PBS). Baseline culture samples were diluted to 10^−5^ and 10^−6^, and stressed culture samples were diluted to 10^−2^ and 10^−3^. These dilutions were then plated 1:10 on TSA for final dilutions of 10^−6/−7^ and 10^−3/−4^ for the baseline culture and stressed culture, respectively. After 3 days incubation (∼25°C), colonies on each plate were enumerated and converted to CFU/ml. For each of the three technical replicates, the total CFUs initially inoculated into the lactic acid media was calculated from the CFU/ml of the baseline culture. Percent survival $( {\frac{{{\mathrm{CFU}}/{\mathrm{ml\ after\ stress}}}}{{{\mathrm{initial\ CFU}}/{\mathrm{ml}}}}} )\ $was calculated for each technical replicate and then averaged to determine the % survival for the biological replicate. This was repeated for a total of six biological replicates. The above protocol was repeated for seven biological replicates of stationary phase cells (OD_600_ ∼1.5) with the modification of using 10^−4/−5^ for the final dilutions of the baseline culture.

### Complementation of *rli47*

For complementation, a construct was generated with *rli47* cloned into the constitutive expression vector pNZ44 (de Ruyter et al. 1996, McGrath et al. 2001). Primers Rli47CompF and Rli47CompR were used to amplify *rli47* from *L. monocytogenes* 6179 genomic DNA using Phusion High-Fidelity DNA Polymerase (Thermo Scientific) (Tables S3 and S4). The resultant amplicon began at the predicted *rli47* TSS and includes the full gene, including the 3′ end that overlaps the terminator of *lmo2412* (Marinho et al. 2019). The amplicon was PCR purified using a PureLink PCR Kit (Invitrogen). The amplicon and pNZ44 were digested with XbaI and HindIII (Thermo Scientific). Both the digested products were purified via gel purification, ligated, and transformed via heat shock into *E. coli* StrataClone as described above. Platinum II Taq Hot-Start DNA Polymerase (Thermo Scientific) was used for subsequent verification steps (Tables S3 and S4). Transformants were verified for the presence of *rli47* in the pNZ44 multiple cloning site using the primers pNZ44F and pNZ44R, and positive colonies were further verified with Sanger sequencing using the same primers by the Iowa State University DNA Facility. The construct was termed p44rli. Electroporation of p44rli into *L. monocytogenes* 6179Δ*rli47* was performed using the same parameters mentioned above. The complement was referred to as 6comprli.

### Flow cytometry to determine if *rli47* affects the number of metabolically active cells following lactic acid exposure

After observing a survival phenotype using the CFU assay, we utilized a staining and flow cytometry approach to assess the proportion of metabolically active 6179, 6179Δ*rli47*, and 6comprli cells before and immediately after lactic acid exposure. Both cells grown to logarithmic and stationary phase were analysed. Cells were grown to the required phase in TSB at 20°C to create a baseline culture. Three samples of 100 μl were removed from the baseline culture and centrifuged for 5 minutes at room temperature at 15 000 × *g*. The supernatant was decanted, and the remainder was then resuspended in PBS for a total of three technical replicates per baseline culture per strain. 100 μl of the baseline culture of each strain was used to inoculate 4900 μl TSB containing 1% (v/v) lactic acid at pH ∼3.4, and the tubes were then incubated at 20°C for 2 hours with 200 rpm shaking. After 2 hours, three samples of 1 ml were harvested from the stressed cells and centrifuged for 5 minutes at 15 000 × *g* for a total of three technical replicates per stressed culture per strain. After decanting the supernatant and resuspending the remainder in 1 ml PBS, the centrifugation, decanting, and resuspension steps were repeated once to further remove any lactic acid.

Cells were prepared for flow cytometry using a BacLight Redox Sensor CTC Vitality Kit (Invitrogen) following the manufacturer’s instructions. For each baseline and stressed technical replicate, 270 μl of cells in PBS were placed in a new tube and incubated for 1 hour at 20°C with 30 μl of 50 mM 5-cyano-2,3-ditolyl tetrazolium chloride (CTC). CTC is a membrane permeable compound that is reduced to a fluorescent derivative by the electron transport chain when absorbed by metabolically active cells, allowing quantification of active cells. After incubation with CTC, cells were fixed by the addition of 30 μl 37% (w/v) paraformaldehyde for a final concentration of 3.36%. Cells were then stained for 30 minutes at 20°C with 1 μl of 3.3 μM SYTO 24, a DNA-binding dye, for a final concentration of 10 nM. Iowa State University Flow Cytometry facility staff performed flow cytometry on stained cells using a FACSCanto (Becton Dickinson) and drew gates using the FACSDiva software v8.0.1 (Becton Dickinson). CTC and SYTO24 were both excited by a 488 nm laser. SYTO24 emission was detected using a 525/50 nm filter, and CTC emission was detected via a 610/20 nm filter. Control samples for gating consisted of *L. monocytogenes* 6179 cells, which were unstained, stained with CTC or SYTO only, and stained with both CTC and SYTO. Gates distinguished cells from debris by the presence of a SYTO signal, and a CTC signal was used to differentiate metabolically active cells from inactive cells. To confirm the validity of the assay, pilot experiments also included cells which had been killed by either heating 80°C for 20 minutes or 3:1 dilution in 70% ethanol. A minimum of three biological replicates were conducted for each strain for each growth phase, and each biological replicate was composed of a minimum of three technical replicates each with a minimum of 3500 cell events. The one exception was a single biological replicate of logarithmic phase 6comprli that had only two technical replicates of lactic acid-exposed cells. The proportion of metabolically active cells was then compared between 6179, 6179Δ*rli47*, and 6comprli.

### Total RNAseq to determine differential gene expression between 6179 and 6179Δ*rli47* under multiple conditions

To further analyse the role of *rli47*, gene expression data were generated for logarithmic *L. monocytogenes* 6179 and 6179Δ*rli47* under multiple conditions: (A) TSB at 37°C [similar to the RNAseq experiment conducted by Marinho et al. (2019) but with a complex media], (B) TSB at 20°C, (C) TSB with 15 mM H_2_O_2_ at 20°C, and (D) TSB with 1% (v/v) lactic acid, pH 3.4 at 20°C. Overnight cultures were generated by adding one colony to 125 μl of TSB and homogenizing with a vortexer. 50 μl of the homogenized colony was then used to inoculate 5 ml of TSB, and cells were grown for 18 hours with 200 rpm shaking at either 20°C or 37°C. Overnight cultures were diluted 1:20 into TSB and subcultured to logarithmic phase (OD_600_ ∼0.4) at either 20°C or 37°C. 1.5 ml of subculture was then inoculated into 6 ml TSB. Tubes for treatments (C) and (D) contained the required H_2_O_2_ or lactic acid concentrations for the final conditions mentioned above. After 30 minutes of 200 rpm shaking at the appropriate temperature, cellular activity was halted by pouring the 7.5 ml of cells into a 15 ml conical tube containing 750 μl of a stop solution (1:10 acid–phenol:chloroform in ethanol) prechilled to −20°C as previously described (Hingston et al. 2017). As in our previous study, 30 minutes was chosen for sampling so as not to cause significant degradation of RNA due to cellular breakdown (Cortes et al. 2020). Tubes were centrifuged at 4696 × *g* for 10 minutes at 0°C, the supernatant was removed, and the pellets were snap-frozen in liquid nitrogen and stored at −80°C.

Conditions (B, C, and D) were also assessed on stationary phase cells, which were prepared in the same manner as in our previous publication (Cortes et al. 2020). Briefly, a large colony of either 6179 or 6179Δrli47 was added to 25 ml of TSB and incubated ∼22 hours at 20°C with 200 rpm shaking. From this culture, a 7.5 ml aliquot was harvested and pelleted at 4696 × *g* for 10 minutes at 20°C; the pellet was then resuspended in 1X PBS and adjusted to an OD_600_ of 3.5. 500 μl of adjusted culture was added to 4.5 ml of TSB (condition B), 4.5 ml of TSB and H_2_O_2_ for a final H_2_O_2_ concentration of 15 mM (condition C), or 4.5 ml TSB and lactic acid for a final lactic acid concentration of 1% (v/v) (condition D). This step was done for three tubes per condition for a total of three technical replicates per biological replicate. After 30 minutes at 20°C with shaking at 200 rpm, all three tubes were pooled and added to a tube of 1.5 ml stop solution. Cells were then pelleted, frozen, and stored as described above.

For RNA extraction, samples were thawed on ice, and 600 μl lysis buffer from the Invitrogen Purelink RNA Mini Kit containing 1% β-mercaptoethanol was added to the tubes. Tubes were then vortexed to resuspend the pellet in lysis buffer. The mixture was then transferred to Lysing Matrix E tubes, and RNA extraction using the Invitrogen Purelink RNA Mini Kit was performed with physical lysis according to the manufacturer’s instructions using a Bead Mill 24 Homogenizer (Fisher Scientific). 1 μl superase inhibitor was added to each sample to prevent RNase activity, and DNA was removed using the Turbo DNA-Free Kit (Thermo Scientific). A PCR targeting the *prfA* gene was performed on the RNA samples to confirm the absence of DNA. RNA quality was validated via the Agilent RNA Nano 6000 Bioanalyzer Assay. All had RNA integrity values ≥8.2 with the exception of one sample from logarithmic phase 6179Δ*rli47* cultured at 37°C that was not measured. The samples were then submitted to the ISU DNA Facility for rRNA depletion, library preparation using an NEBNext Ultra II Directional RNA Library Prep Kit (New England BioLabs), and single-end stranded sequencing utilizing the Illumina NovaSeq 6000 platform (100 cycles using an SP flow cell).

### Data analysis

Raw data from the growth rate, CFU, and flow cytometry experiments were processed and organized in Microsoft Excel. Growth rates and associated metrics were calculated from raw data using the command line program GrowthRates (v4.41) (Hall et al. 2014). For flow cytometry data, the ratios between metabolically active cells in the stressed and control experiments $( {\frac{{{\mathrm{\% \ active\ cells\ in\ stressed\ population}}}}{{{\mathrm{\% \ active\ cells\ in\ control\ population}}}}} )\ $ were converted into log2fold changes. Statistical analysis was performed in R (v4.2.1). QQplots were generated to assess normality, and Bartlett’s test was performed to analyse the homoscedasticity of data between groups. Student’s *t*-test was used to test for equality of means for two-sample pairwise comparisons, where data were homoscedastic; in the cases where data were heteroscedastic, Welch’s *t*-test was used instead. One-way ANOVA was used to test for differences between the means of >2 groups, where data met the assumptions of normality and homoscedasticity.

For RNAseq analysis, raw reads were inspected for quality with FASTQC (v0.11.7) (Andrews 2010). Trimming of bases <Q30, trimming of Illumina adapter sequences, and filtering of reads that were shortened below 75 bp by trimming was performed in BBDuk (BBMap v39.01) using the following options: ktrim=r, ordered, k=23, hdist=1, mink=11, qtrim=rl, trimq=30, maq=30, and minlen=75 (Bushnell 2024). FASTQC was then utilized to confirm removal of adapter sequences. Read mapping against the *L. monocytogenes* 6179 genome (chromosome and plasmid) (GenBank accessions CP098509.1 and CP098510.1) was performed with BowTie2 within the RNA-seq by expectation maximization (RSEM) software (v1.3.3) (Li and Dewey 2011, Tibbs-Cortes et al. 2023). RSEM was used to perform transcript quantification using the *L. monocytogenes* 6179 GTF file modified to include an annotation for *rli47* from bases 1 748 283 to 1 748 797 on the positive strand of the chromosome, and the read start position distribution (RSPD) option was enabled to allow modeling to account for read start position distribution biases.

RSEM estimated counts per gene were imported into R (v4.4.0) using the tximport package (Soneson et al. 2016). Notably, RSEM will assign an effective length of 0 to genes where no reads mapped. To prevent this from causing errors in downstream processing, genes with an effective length of 0 were given an effective length of 1 as recommended by (Love 2021). Differential gene expression analysis was then performed on the RSEM estimated counts using DESeq2 (v1.44.0) with multifactorial design to account for batch effects arising from different biological replicates and different RNA extraction dates. DESeq2 calculates adjusted *P*-values (*q*-values) using the Benjamini and Hochberg method to account for multiple testing correction (Love et al. 2014). Genes with a *q*-value <0.05 were considered differentially expressed.

### Comparative genomics of *rli47* across *L. monocytogenes* strains

All complete *L. monocytogenes* genomes available in the NCBI RefSeq database (*n* = 506 as of 25 September 2025) were downloaded via the NCBI Datasets CLI (v18.8.0) and used to create a local BLAST database. BLASTn with -max_hsps 1 and -word_size 6 was used to query the *L. monocytogenes* EGD-e *rli47* gene (from the TSS to the predicted end of the *rli47* transcript) against the strains in the local database.

### Modification of CopraRNA, an *in silico* sRNA target identification program, for application to Rli47

Direct interaction between an sRNA and an mRNA molecule is the typical mechanism for sRNA-mediated gene regulation in bacteria (Lybecker et al. 2014), and *in silico* tools enable the identification of putative sRNA targets by modeling thermodynamic interactions between an sRNA and transcripts (Georg et al. 2020). Previously, the program IntaRNA was used to identify the *ilvA* transcript as a potential target for Rli47 binding prior to functional characterization of Rli47 (Marinho et al. 2019). Here, we utilized CopraRNA to complement RNA sequencing analysis and identify potential alternative targets of Rli47. CopraRNA leverages comparative genomics to hone sRNA target prediction, assuming that if an sRNA is conserved among species then its target(s) are likely conserved as well. IntaRNA predictions are performed for multiple species, and the relatedness of the species and consensus of predicted targets are used to identify likely sRNA targets (Wright et al. 2013, 2014, Mann et al. 2017).

However, in its original implementation CopraRNA cannot be applied to strains which are not part of the RefSeq database (O’Leary et al. 2016). Additionally, although the underlying IntaRNA program can accept user-provided temperature values to improve its predictions, these values and other options cannot be passed by the user through CopraRNA to the underlying IntaRNA runs. We modified the source code of CopraRNA (v2.1.3) (https://github.com/PatrickRWright/CopraRNA) to accept any prokaryotic genome that is available in the NCBI Genomes FTP site (https://ftp.ncbi.nlm.nih.gov/genomes/GENOME_REPORTS/prokaryotes.txt), expanding the list of genomes usable by CopraRNA beyond only RefSeq genomes as originally implemented. We also added code to permit the usage of any IntaRNA option in the initial CopraRNA call. Running the modified software on example data provided on the CopraRNA webserver (rna.informatik.uni-freiburg.de/CopraRNA/Input.jsp) demonstrated that the modified software produced the same results as the unmodified software.

BLASTn was utilized as described above to acquire the sequences of *rli47* homologs from seven *Listeria sensu stricto* strains with whole genomes available on NCBI (Orsi et al. 2023): *L. monocytogenes* 6179 (NZ_HG813249), *L. monocytogenes* EGD-e (NC_003210), *L. monocytogenes* 10403S (NC_017544), *L. seeligeri* SLCC3954 (NC_013891), *L. ivanovii* subsp. *ivanovii* WSLC3010 (NZ_CP009577), *L. innocua* CFSAN044836 (NZ_CP045743), and *L. welshimeri* NCTC11857 (NZ_LT906444). Genome version NZ_HG813249 was used for *L. monocytogenes* 6179 because the newer version, CP098509 was not yet available on the NCBI Genomes FTP site. The *rli47* homologs and genomes of these strains were used as the input for CopraRNA, and putative targets were searched for among regions spanning 100 bp up and downstream of all annotated coding sequences (CDS). To evaluate putative Rli47 targets across all genomes analysed, no organism of interest or consensus was used in the aggregation of *P*-values by CopraRNA (–nooi, –cons=0). Additional IntaRNA parameters included: –outOverlap=Q –seedBP=7, –outDeltaE=100, and either –temperature=37 or 20. For each strain, the top two most likely interactions predicted by IntaRNA (in terms of free energy) were reported for every region. Because the region encompassing the 3′ end and 100 bp downstream of *lmo2142* is directly antisense to the *rli47* locus, any predicted interactions between the end of *lmo2142* with Rli47 were ignored. MAFFT (v7.505) was used to align the loci of *rli47* (bases 1–515 beginning at the TSS) and *ilvA* (bases −100–1369 relative to the beginning of the CDS) using the L-INS-i method with 1000 iterations (Katoh and Standley 2013). Alignments of *rli47* (Supplementary File 5) and *ilvA* (Supplementary File 6) were visualized using Jalview (v2.11.4.0) (Waterhouse et al. 2009).

## Results

### 6179 and 6179Δ*rli47* do not display different growth metrics when grown in TSB at 37°C or 25°C

**Figure 1.**
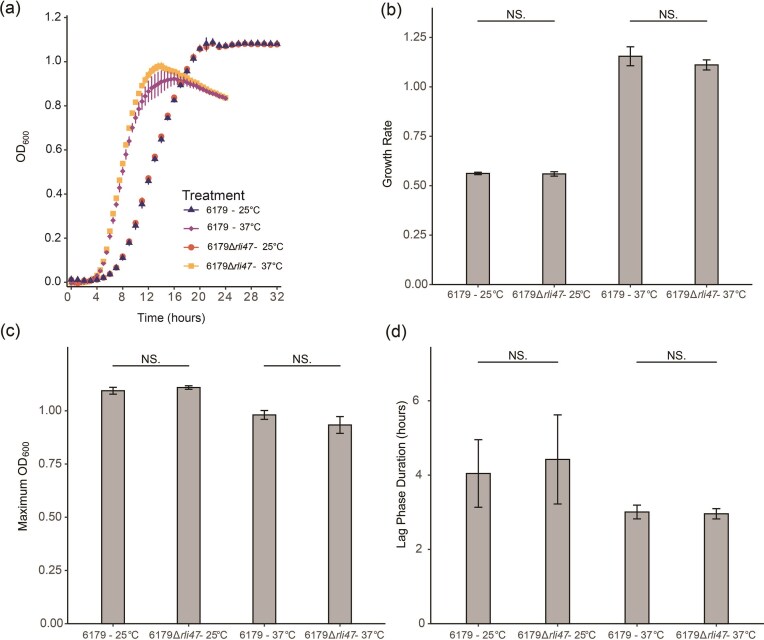
Comparison of growth rates and associated metrics between 6179 and 6179Δ*rli47* grown at 25°C in TSB. Error bars represent standard deviation. (A) Representative growth curves of 6179 and 6179Δ*rli47* constructed from the average OD_600_ of three biological replicates with four technical replicates each. (B) Growth rates of 6179 and 6179Δ*rli47*. Results represent averages of three biological replicates. (C) Maximum OD_600_ values of 6179 and 6179Δ*rli47*. Results represent averages of three biological replicates. (D) Lag phase duration of 6179 and 6179Δ*rli47*. Results represent averages of three biological replicates. NS: *P* > .05 by Welch’s *t*-test.

To determine if deletion of *rli47* affected growth of *L. monocytogenes* strain 6179 when grown in TSB, wild type 6179 and its constructed isogenic *rli47* deletion mutant, 6179Δ*rli47*, were grown in TSB at both 37°C and 25°C. Representative growth curves were constructed from the average OD_600_ values for each biological replicate (Fig. 1A). Hourly OD_600_ measurements were utilized by the program GrowthRates to calculate the average growth rate (Fig. 1B), maximum OD_600_ value (Fig. 1C), and lag phase durations (Fig. 1D) for each strain. For each of these growth metrics, the mean values were not significantly different between 6179 and 6179Δ*rli47* at either temperature (Student’s *t*-test, *P* > .05).

### 6179Δrli47 exhibits higher lactic acid survival than 6179 at logarithmic but not at stationary phase

**Figure 2.**
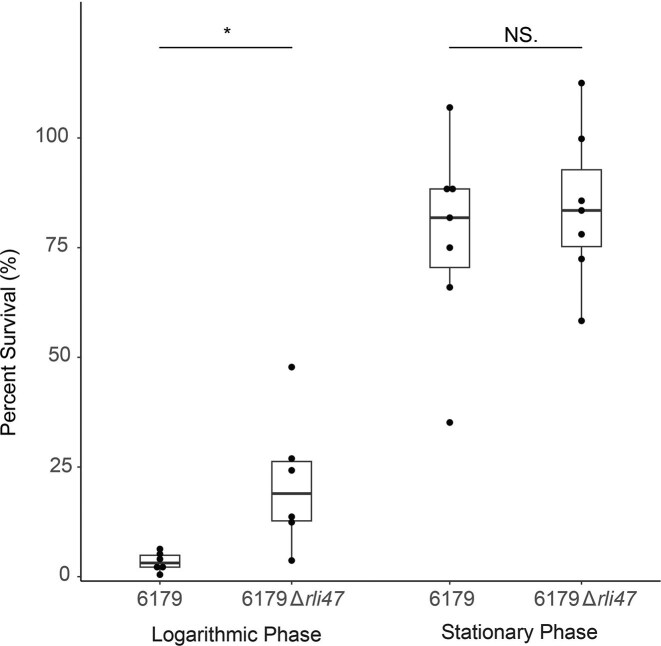
Percent survival of 6179 and 6179Δ*rli47*. Percent survival of logarithmic (top) and stationary (bottom) phase 6179 and 6179Δ*rli47* following a 2-hour exposure to 1% lactic acid, pH 3.4–3.6, at 20°C. Points represent the average % survival of three technical replicates. Logarithmic phase means were compared using Welch’s *t*-test due to heteroscedasticity. Stationary phase means were homoscedastic and compared by Student’s *t*-test. * = *P* < .05.

To investigate the role of *rli47* in lactic acid stress response, a CFU approach was utilized to compare the % survival of 6179 and 6179Δ*rli47* after 2-hour exposure to 1% lactic acid pH 3.4–3.6. The results of the experiments demonstrated that the average % survival of logarithmic phase 6179 (3.4%) was significantly lower (Welch’s *t*-test, *P* = .035) than that of logarithmic phase 6179Δ*rli47* (21.4%) (Fig. 2). In contrast, no significant difference (Student’s *t*-test, *P* = .53) was observed between the average % survivals of either strain at stationary phase (6179–77.4%, 6179Δ*rli47–*84.3%).

### Flow cytometry reveals no difference between 6179 and 6179Δ*rli47* in terms of the change in proportion of metabolically active cells following lactic acid stress

**Figure 3.**
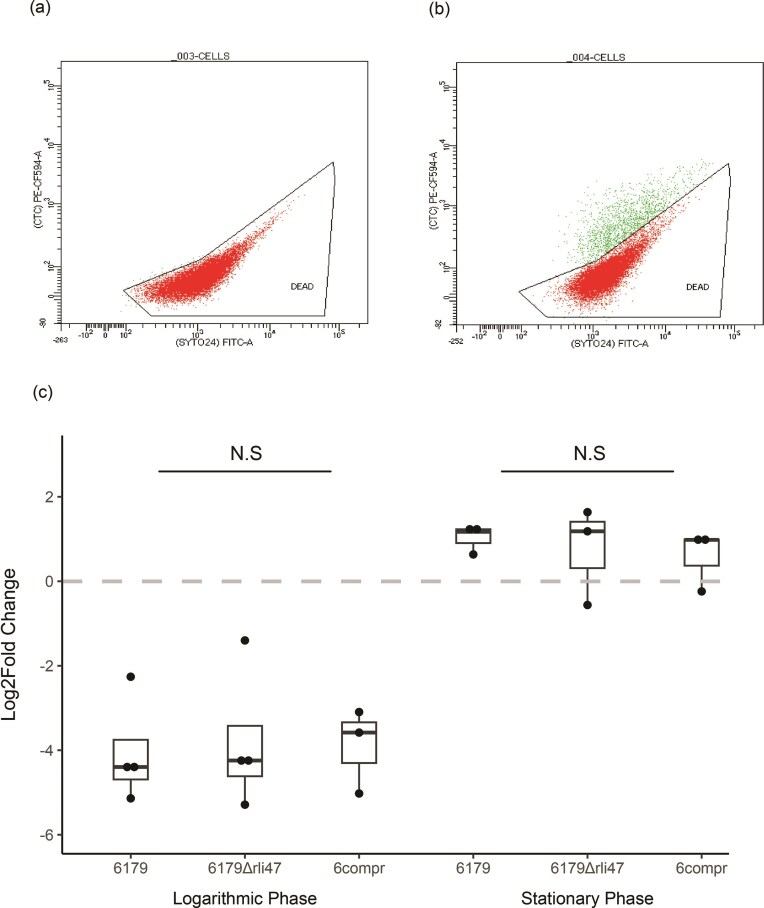
Comparison of log2fold changes in the proportion of metabolically active cells following lactic acid stress. Individual points represent a single biological replicate. N.S. = not significant. (A) Flow cytometry output for 6179 diluted 3:1 in 70% ethanol prior to CTC staining. (B) Flow cytometry output for 6179 grown to logarithmic phase (untreated control). (C) Analysis of the mean log2fold changes of strains grown to logarithmic and stationary phases (one-way ANOVA).

A flow cytometry approach to quantify metabolically active cells was used to further characterize the lactic acid survival phenotype observed in the CFU assays. Pilot flow cytometry experiments demonstrated that both metabolically active and inactive *L. monocytogenes* cells could be detected by CTC and SYTO staining (Fig. 3A and B). The proportion of metabolically active cells was measured before and after 2-hour exposure to 1% lactic acid for 6179, 6179Δ*rli47*, and 6comprli. For all three strains grown to logarithmic phase, exposure to lactic acid resulted in a decrease (negative log2fold change) in the percentage of metabolically active cells. The opposite effect was observed for cells grown to stationary phase; after challenge with lactic acid, the proportion of metabolically active cells of all three strains exhibited log2fold increases of ∼1. No significant differences between the mean log2fold changes of all three strains were detected for logarithmic or stationary phase cells (one-way ANOVA, *P* > .05) (Fig. 3C).

### RNAseq reveals minimal differences in the transcriptomes of 6179 and 6179Δ*rli47*

RNAseq was utilized to identify differences in gene expression between 6179 and 6179Δ*rli47* that might account for the increased lactic acid survival of 6179Δ*rli47*. The transcriptomes of logarithmic phase 6179 and 6179Δ*rli47* were compared under four conditions: (A) TSB at 37°C [comparable to the transcriptome analysis conducted by Marinho et al. (2019)], (B) TSB at 20°C, (C) TSB with 15 mM H_2_O_2_ at 20°C, and (D) TSB with 1% (v/v) lactic acid, pH 3.4 at 20°C. Conditions (B, C, and D) were also investigated for stationary phase cells. Reads mapping to *rli47* were detectable in both logarithmic and stationary phase wild type 6179 cells under all conditions, although more reads were present in stationary phase cells than logarithmic phase cells under conditions (B, C, and D). As expected, *rli47* was significantly downregulated in 6179Δ*rli47* compared to the wild type under all conditions at both growth phases with a log2fold change ranging from −8.74 to −20.49 (Table 2, Supplementary File 7).

**Table 2.** Differentially expressed genes between 6179 and 6179Δ*rli47* per growth phase and condition.

Growth phase	Condition	Gene	log2FoldChange^a^	*q*-value
Logarithmic	37°C	*rli47*	−11.14	0.000
Logarithmic	20°C	*rli47*	−11.19	0.000
Stationary	20°C	*rli47*	−10.25	0.007
		*lmo2361* (*LM6179_03079*)	−1.07	0.007
		*lmo2360* (*LM6179_03078*)	−0.97	0.021
Logarithmic	15 mM H_2_O_2_, 20°C	*rli47*	−8.74	0.000
Stationary	15 mM H_2_O_2_, 20°C	*rli47*	−13.42	0.000
		*lmo2115* (*LM6179_02890*)	−0.89	0.004
		*lmo2114* (*LM6179_02889*)	−0.81	0.038
Logarithmic	1% lactic acid, 20°C	*rli47*	−10.22	0.000
Stationary	1% lactic acid, 20°C	*lmo2143* (*LM6179_02919*)	3.16	0.000
		*rli47*	−20.49	0.000

^a^Positive log2foldchange values are reported for 6179Δ*rli47* relative to 6179 (e.g. a negative log2foldchange value indicates that a gene is downregulated in 6179Δ*rli47* relative to the wild type).

For logarithmic phase cells, no genes besides *rli47* were differentially expressed between 6179Δ*rli47* and 6179 in any of the four conditions. In contrast, differential expression of additional genes besides *rli47* was observed for stationary phase cells exposed to conditions (B, C, and D). The genes *LM6179_03078* (corresponding to *lmo2360* in *L. monocytogenes* EGD-e and encoding an uncharacterized protein) and *LM6179_03079* (corresponding to *lmo2361* and encoding a putative transcriptional regulator) were both downregulated in 6179Δ*rli47* at 20°C by a log2fold change of ∼1 compared to the wild type. A similar downregulation of approximately log2fold 0.9 was observed in 6179Δ*rli47* in condition C for *LM6179_02889* and *LM6179_2890* (*lmo2114*/*anrA* and *lmo2115*/*anrB*, respectively), which encode components of a putative multidrug resistance transporter. Finally, following exposure to lactic acid in condition D, *LM6179_02919* (*lmo2143*, encoding a mannose-6-phosphate isomerase) was upregulated in 6179Δ*rli47* compared to the wild type (Table 2).

### *rli47* is ubiquitous across strains of *L. monocytogenes*

BLASTn identified a homolog of *rli47* in all 506 *L. monocytogenes* genomes found in RefSeq. All homologs were within 5 nt in length compared to the EGD-e *rli47* gene, and the minimum % nucleotide identity relative to that of EGD-e *rli47* was 96.62%. All BLASTn results can be found in Supplementary File 8.

### CopraRNA predicts putative mRNA targets of Rli47

CopraRNA predictions for Rli47 at 20°C and 37°C are listed in Table 3 along with the underlying IntaRNA results for 6179. Complete CopraRNA results and IntaRNA results for all strains at 20°C and 37°C are found in Supplementary Files 9 and 10. Transcripts of *lmo2078* (*tsaE*), *lmo0389* (*ltrA*), and *lmo2630* (*rplW*) were predicted to interact with Rli47 at both temperatures; the predicted Rli47-*tsaE* interaction was the most favorable interaction at both 20°C and 37°C. Notably, the *tsaE*-Rli47 interaction predicted by IntaRNA involved the Shine–Dalgarno sequence of *tsaE* and the CU-rich third stem loop of Rli47, similar to the experimentally verified interaction of Rli47 with the *ilvA* transcript. This interaction of *tsaE* and Rli47 was predicted for all strains at either temperature with the exception of *L. ivanovii* at 37°C, where the *tsaE* Shine–Dalgarno region was more likely to interact with the fourth Rli47 stem loop structure, stem loop D. At 37°C, *lmo1677* (*menA*) and *lmo1894* (*nth*) were also predicted to interact with Rli47. The interaction of Rli47 with the *lmo2629* (*rplB*) 5′ CDS was also predicted at 37°C; the region of the genome encoding the 5′ CDS of *rplB* is the same region encoding the 3′ untranslated region of *rplW* due to the proximity of the two genes in the genome.

**Table 3.** Putative targets (FDR < 0.05) for Rli47 based on CopraRNA as well as CopraRNA metrics for the canonical *ilvA*-Rli47 interaction.

Temperature	False discovery rate	EGD-e locus tag	6179 locus tag	Gene	Energy (kcal/mol)	CDS length in 6179	mRNA interaction site start in 6179	mRNA interaction site end in 6179	Rli47 interaction site start in 6179	Rli47 interaction site end in 6179	Annotation
20	0.000	*lmo2078*	*lm6179_rs13980*	*tsaE*	−38.05	462	−40	9	195	242	tRNA (adenosine(37)-N6)-threonylcarbamoyltransferase complex ATPase subunit type 1 TsaE
	0.002	*lmo0389*	*lm6179_rs03370*	*ltrA*	−27.88	1116	953	999	434	477	Low temperature requirement protein A
	0.010	*lmo2630*	*lm6179_rs00205*	*rplW*	−23.87	285	339	375	70	106	50S ribosomal protein L23
	0.258	*lmo1991*	*lm6179_rs13545*	*ilvA*	−16.53	1268	212	235	378	401	Threonine ammonia-lyase
37	0.000	*lmo2078*	*lm6179_rs13980*	*tsaE*	−30.71	462	−40	9	195	242	tRNA (adenosine(37)-N6)-Threonylcarbamoyltransferase complex ATPase subunit type 1 TsaE
	0.001	*lmo0389*	*lm6179_rs03370*	*ltrA*	−23.56	1116	953	999	434	477	Low temperature requirement protein A
	0.008	*lmo2630*	*lm6179_rs00205*	*rplW*	−19.49	285	347	375	70	98	50S ribosomal protein L23
	0.028†	*lmo2629*	*lm6179_rs00200*	*rplB*	−17.95	834	22	50	70	98	50S ribosomal protein L2
	0.039	*lmo1677*	*lm6179_rs11840*	*menA*	−18.45	960	388	420	133	163	1 4-dihydroxy-2-naphthoate polyprenyltransferase
	0.042	*lmo1894*	*lm6179_rs13060*	*nth*	−17.56	660	310	357	125	169	Endonuclease III
	0.292	*lmo1991*	*lm6179_rs13545*	*ilvA*	−11.95	1268	−11	1	229	241	Threonine ammonia-lyase

mRNA interaction site coordinates are relative to the beginning of the CDS (position 1). †The 3′ untranslated region of *rplW* and the beginning of the *rplB* CDS that are most likely to interact with Rli47 are the same sequence in the 6179 genome due to the proximity of the two genes.

CopraRNA did not predict *ilvA* to be a target for Rli47 at either temperature. The IntaRNA results underlying CopraRNA showed that at 37°C, the canonical interaction of Rli47 stem loop three with the *ilvA* transcript Shine–Dalgarno sequence was one of the two most likely interactions predicted for all *L. monocytogenes* strains. Interaction of the *ilvA* Shine–Dalgarno sequence with Rli47 at 37°C was also predicted in *L. innocua, L. seeligeri, L. welshimeri*, but not *L. ivanovii*, although the predicted interacting regions were longer than the canonical interaction in *L. monocytogenes* (Supplementary File 10). At 20°C, interaction of Rli47 stem loop three with the *ilvA* Shine–Dalgarno region was predicted by IntaRNA only in *L. monocytogenes* 6179, *L. seeligeri, L. innocua*, and *L. welshimeri* (Supplementary File 9).

## Discussion

RLi47 is known to function in suppression of isoleucine biosynthesis via direct binding to the Shine–Dalgarno region of the *ilvA* transcript, potentially to confer an advantage to *L. monocytogenes* by limiting cellular growth under adverse conditions (Marinho et al. 2019). However, previous experiments testing the role of *rli47* in the *L. monocytogenes* response to oxidative and inorganic acid stress did not identify a difference in phenotype between an isogenic *rli47* deletion mutant and its parent (Mujahid et al. 2013). Transcriptomic analyses have in contrast revealed significant upregulation and high transcription levels of *rli47* in response to lactic acid (Cortes et al. 2020), coculture (Anast and Schmitz-Esser 2020), and high pressure processing (Duru et al. 2021). Following the upregulation of *rli47* in our previous study, we sought to investigate the role of *rli47* in *L. monocytogenes* exposed to oxidative and pH stress at temperatures relevant to food production. Because *rli47* is also expressed in logarithmic phase (albeit to a lesser extent than stationary phase) (Mujahid et al. 2013, Marinho et al. 2019), we also analysed cells in exponential growth.

### Deletion of *rli47* does not result in a growth phenotype in media containing isoleucine

We observed no significant difference between the growth kinetics of 6179 and 6179Δ*rli47* in terms of growth rate, maximum OD, or lag phase duration at either 37°C or 25°C in TSB (Fig. 1). Similarly, no differences in growth kinetics were observed between *L. monocytogenes* 10403S and 10403SΔ*rli47* grown in BHI at 7°C or between *L. monocytogenes* EDG-e and EGD-eΔ*rli47* grown in a defined media at 37°C (Mujahid et al. 2013, Marinho et al. 2019). It was previously demonstrated, however, that when isoleucine was excluded from the same defined media, EGD-eΔ*rli47* exhibited a shorter lag phase at 37°C (Marinho et al. 2019). While it seems likely that the isoleucine present in both TSB and BHI explains the absence of a growth phenotype in 6179Δ*rli47* and 10403SΔ*rli47* (Wretlind 1947, Zhu et al. 2005), further growth experiments testing the kinetics of these *rli47* deletion mutants with and without isoleucine would be necessary to definitively test this hypothesis.

### 6179Δrli47 exhibits higher lactic acid survival at logarithmic growth phase but not higher metabolic activity

We had previously observed that reads mapping to *rli47* comprised 28% of the entire 6179 transcriptome following lactic acid stress (Cortes et al. 2020); similarly, transcriptome analysis of *L. monocytogenes* RO15 and ScottA following high pressure processing determined that an average of ∼53% and ∼28% of the reads in the respective transcriptomes mapped to *rli47*, respectively (Duru et al. 2021). The tremendous expression level of *rli47* under these conditions and its transcriptional control by σ^B^ led us to hypothesize that 6179Δ*rli47* would demonstrate severely attenuated survival after lactic acid exposure compared to the wild type. Intriguingly, the CFU assays demonstrated no difference between the survival of stationary phase 6179 and 6179Δ*rli47* when exposed to lactic acid, but 6179Δ*rli47* exhibited a % survival over 8 times greater than that of 6179 at the logarithmic phase (Fig. 2).

To investigate this phenotype further, we performed flow cytometry on both logarithmic and stationary phase 6179, 6179Δ*rli47*, and 6comprli (Fig. 3). As expected, stationary phase cells of all three strains exhibited higher acid tolerance than logarithmic phase cells as demonstrated by the higher proportion of metabolically active cells. There was no significant difference between log2fold changes (before versus after lactic acid exposure) of metabolically active 6179, 6179Δ*rli47*, and 6comprli cells at either logarithmic or stationary phase. A higher proportion of sublethally injured cells among the metabolically inactive 6179Δ*rli47* population could explain the observation of a survival phenotype in the absence of a change in the proportion of metabolically active cells between 6179 and 6179Δ*rli47*. It is conceivable that the absence of *rli47* could somehow result in a reduction of lethal injury from lactic acid, thereby resulting in a larger population of sublethally injured cells in 6179Δ*rli47* compared to 6179. These sublethally injured cells would eventually recover and be detected by the CFU approach, displaying the difference in overall survival, but they would not immediately resume metabolic activity, thereby being undetectable by our CTC staining approach. In support of this hypothesis, sublethally injured *L. monocytogenes* have been detected after organic acid stress (Arvaniti et al. 2021). Future research should repeat the survival assay and flow cytometry experiments at additional timepoints to gain a more comprehensive understanding of the survival and metabolic activity kinetics of 6179 and 6179Δ*rli47*.

The higher survival exhibited by 6179Δ*rli47* may be explained by the relationship between the *L. monocytogenes* membrane, organic acid stress, and *rli47*. Compared to inorganic acids, organic acids are generally more harmful to bacteria due to their capacity to diffuse across the cell membrane while in their uncharged, undissociated state. Subsequent dissociation of an organic acid within a cell then causes a complicated array of negative effects on cell physiology including the disruption of membrane integrity (Cotter and Hill 2003, Hirshfield et al. 2003, Lund et al. 2014, 2020). As a response to this disruption of membrane integrity *L. monocytogenes* has been shown to modulate its membrane composition in response to organic acid stress. Exposure of logarithmic phase *L. monocytogenes* cells to organic acid resulted in higher proportions of anteiso-branched chain fatty acids (BCFAs) compared to cells grown under control conditions (Giotis et al. 2007). Isoleucine is a precursor for anteiso-BCFAs (Sun et al. 2012), and therefore it is plausible that *rli47* expression could negatively regulate anteiso-BCFAs in the *L. monocytogenes* membrane by suppression of isoleucine biosynthesis. If this is the case, absence of Rli47 could result in an increased level of anteiso-BCFAs in the 6179Δ*rli47* membrane, possibly conferring a higher degree of organic acid tolerance. Additionally, the mechanistic differences between strong acid stress and organic acid stress may partially explain why no phenotype was observed for *rli47* in previous studies on its role in inorganic acid stress (Mujahid et al. 2013).

### Differences in gene expression between 6179 and 6179Δ*rli47* are only observed at stationary phase

RNA sequencing was performed to gather additional insight into the function of Rli47 and to complement the CFU and flow cytometry experiments. Transcriptomic analyses of *rli47* deletion mutants have been performed previously with vastly different results. When *L. monocytogenes* EGD-eΔ*rli47* was grown to logarithmic phase in a defined media at 37°C, over 155 genes were differentially expressed compared to the wild type (Marinho et al. 2019). In contrast, only two genes, *lmo0636* and *lmo0637*, were differentially expressed in a 10403SΔ*rli47* mutant grown to stationary phase in BHI at 37°C (Mujahid et al. 2013). Notably, *lmo0636* and *lmo0637* were not among the differentially expressed genes identified in the experiment by Marinho et al. (2019). Differences in strain, the media used, and growth phase of the cells all could have driven the disparity between these two Δ*rli47* transcriptomes. In this study, the transcriptomes of logarithmic phase 6179 and 6179Δ*rli47* were compared under four conditions: TSB at 37°C, TSB at 20°C, TSB at 20°C with 15 mM H_2_O_2_, and TSB at 20°C with 1% (v/v) lactic acid, pH 3.4 (conditions A, B, C, and D respectively). Additionally, due to the previously demonstrated differences in *rli47* expression at stationary phase, we analyzed stationary phase 6179 and 6179Δ*rli47* in conditions (B, C, and D).

Strikingly, we did not observe any differential expression of genes (besides *rli47*) in any of the four conditions at logarithmic phase. This was especially intriguing for condition D, as the absence of differential expression between 6179 and 6179Δ*rli47* is difficult to reconcile with the 6179Δ*rli47* lactic acid survival phenotype observed in logarithmic phase cells. This observation could be explained by the use of different lactic acid exposure durations for the RNAseq (30 minutes) and CFU experiments (2 hours). It is possible that any differential gene expression underlying the survival difference between logarithmic phase 6179 and 6179Δ*rli47* does not occur at 30 minutes post-stress induction. For example, Rli47-induced transcriptomic changes could have occurred early during the subculture step prior to exposure to lactic acid, altering membrane physiology or somehow else priming *L. monocytogenes* against organic acid stress. Alternatively, Rli47 may act in a manner in this scenario that cannot be detected by gene expression analysis. Rli47 could potentially affect DNA superstructures through interaction with nucleoids as has been shown for other noncoding RNAs (Lybecker et al. 2014). Alternatively, Rli47 could interact with proteins or regulate gene expression posttranscriptionally (Storz et al. 2011, Mellin and Cossart 2012, Dutta and Srivastava 2018).

Our findings for logarithmic phase cells grown under condition A were also surprising, as the absence of differential expression starkly contrasted the findings of (Marinho et al. 2019) where the EGD-e *rli47* deletion mutant differentially expressed 155 genes. While our study analysed a different strain of *L. monocytogenes*, we would have expected to see at least some overlap in the responses between strains, and the growth phase and temperature of condition A are the same as those tested by Marinho et al. (2019). Therefore, it seems likely that the primary driver of difference between the two RNAseq experiments is the use of a complex, rich media (TSB) in our analysis compared to the less complex, defined chemical medium used by Marinho et al. (2019). Despite their differences, the media of both experiments contain isoleucine, and so the disparity in transcriptomes strongly indicates that exogenous molecules other than isoleucine and/or other conditions unique to growth in TSB may affect Rli47 activity and function. Based on our results as well as the results from *L. monocytogenes* grown in BHI (Mujahid et al. 2013), it may be that Rli47 does not alter the transcriptome of *L. monocytogenes* during growth in rich media at 37°C. Similarly, Rli47 does not alter *L. monocytogenes* transcription under the other tested conditions during logarithmic phase. The results from 6179 and 6179Δ*rli47* grown in all conditions at logarithmic phase support previous findings that *rli47* is transcribed during exponential growth; whether or not this transcription is biologically relevant for *L. monocytogenes* at logarithmic phase remains to be demonstrated.

While no differential gene expression was observed between logarithmic phase 6179 and 6179Δ*rli47*, some differential gene expression was observed between stationary phase cultures. This was again unexpected as no survival phenotype was observed between 6179 and 6179Δ*rli47* at stationary phase. At growth in TSB at 20°C (condition B), *lmo2360* and *lmo2361* were both downregulated ~2-fold in 6179Δ*rli47* (Table 2). Lmo2360 is an uncharacterized protein with a similar domain to a surface protein in *Lactococcus lactis* that is necessary for infection of *L. lactis* by certain phages. Lmo2361 is annotated as an Rrf2 transcriptional regulator, a widespread class of transcriptional regulators with helix-turn-helix DNA-binding domains (Shepard et al. 2011). *Lmo2361* is itself under the control of the transcription factor GadR, a major positive regulator of the acid tolerant response whereby exposure to mild acidic conditions triggers transcriptional changes that result in a higher resistance to strong acid conditions (Wu et al. 2023). The function of Lmo2361 is unknown, although a transposon mutant exhibited slightly decreased hemolysis (Zemansky et al. 2009). As neither protein has been characterized in *L. monocytogenes*, future research will be necessary to determine if the relationships between *rli47* and *lmo2360* and *lmo2361* are biologically relevant.

In response to H_2_O_2_, the genes *anrA* (*lmo2114*) and *anrB* (*lmo2115*) were both slightly downregulated (log2fold changes of −0.81 and −0.89, respectively) in 6179Δ*rli47* compared to the wild type. These genes encode the ATP binding component and permease of the ATP synthase binding cassette (ABC) transporter AnrAB, a multidrug resistance transporter that imparts resistance to antibiotics that target the cell wall and membrane including nisin, bacitracin, and β-lactams (Collins et al. 2010). Multidrug resistance ABC transporters have been observed to be important for oxidative stress *in vitro* for bacteria including *Serratia marcescens* and *Salmonella enterica* serovar Typhimurium. In particular, an ABC transporter of *S. enterica* sv. Typhimurium was required for the pathogen to survive reactive oxygen species during growth in murine macrophages (Bogomolnaya et al. 2013, Shirshikova Tatiana et al. 2021). Supporting the idea that AnrAB is important in the *L. monocytogenes* response to oxidative stress, both *anrA* and *anrB* are also known to be upregulated in mouse tissues (Camejo et al. 2009) and in murine macrophages (Schultze et al. 2015) during *L. monocytogenes* infection. In this model, then, Rli47 is somehow involved in increasing transcription of *anrAB*. However, although *anrAB* was more expressed in 6179 in condition C compared to the *rli47* deletion mutant, comparing growth of stationary phase cells at 20°C compared to growth in 15 mM H_2_O_2_ revealed that both genes were significantly downregulated in both strains following H_2_O_2_ stress: log2fold changes for *anrA* and *anrB* were −1.68 and −1.94, respectively, in 6179 and −2.22 and −2.49, respectively, in 6179Δ*rli47*. This would indicate that expression of *anrAB* is not advantageous in the response to oxidative stress caused by H_2_O_2_. Further research is therefore necessary to determine the role of *anrAB* in the *L. monocytogenes* oxidative stress response and the role, if any, that Rli47 plays in regulating *anrAB* expression.

The combination of stationary phase cells and the parameters of condition D were done to replicate our previous study, where we observed that an average of 28% of all sequenced reads mapped to *rli47* following exposure of 6179 to 1% lactic acid for 30 minutes at 20°C. We found that reads mapping to *rli47* accounted for an average of 4.79% of all reads in the 6179 lactic acid replicates. Although this is much lower than the 28% of reads observed previously, *rli47* was the second most transcribed gene in the 6179 lactic acid transcriptome after the transfer–messenger RNA gene *ssrA*. The differences here could be due to different ribosomal RNA depletions and other library preparation steps between the two experiments.

Because of the large number of *rli47* transcripts in this previous experiment, we expected to observe multiple differentially expressed genes between 6179 and 6179Δ*rli47*. Even if *rli47* only directly affected the transcription of a relatively small number of genes, we expected that reduced transcriptomic burden in 6179Δ*rli47* would result in other genes being transcribed at different levels (Price et al. 2016). Contrary to our hypothesis only the gene *lmo2143* was differentially expressed in the deletion mutant (log2fold change of 3.16) besides *rli47*. Lmo2143 is annotated as a mannose-6-phosphate isomerase with homology to the mannose-6-phosphate isomerase of *Bacillus subtilis*, ManA. ManA can isomerize a number of aldoses and ketoses and is important for metabolism as well as proper maintenance of cell wall structure and its carbohydrate composition (Yeom et al. 2009, Elbaz and Ben-Yehuda 2010). If Lmo2143 is similarly involved in cell wall homeostasis, then it is possible that its activity might be modulated directly or indirectly by Rli47 to counter cell wall perturbations induced by organic acid stress (Mols et al. 2010, Manzo et al. 2021, Panda et al. 2024). In support of this hypothesis, *lmo2143* was previously observed to be upregulated in response to lactic acid (Cortes et al. 2020), suggesting that further upregulation of this gene in the absence of Rli47 would be advantageous under lactic acid stress.

### Rli47 is predicted to interact with additional RNA transcripts besides that of *ilvA*

Application of CopraRNA suggested that Rli47 could potentially have regulatory roles in addition to its known role in isoleucine biosynthesis. CopraRNA predicted interactions between Rli47 and three transcripts at both 20°C and 37°C: *lmo2078* (*tsaE*), *lmo0389* (*ltrA*), and *lmo2630* (*rplW*). The genes *lmo1677* (*menA*), *lmo2629* (*rplB*), and *lmo1894* (*nth*) were also identified as putative targets for RLi47 at 37°C. None of the putative targets were differentially expressed in any of our experiments or previous analyses of *rli47* deletion mutants (Mujahid et al. 2013, Marinho et al. 2019). It is possible that these putative interactions do not actually occur or that any biological function resulting from these predicted interactions is not detectable by transcriptome analysis as discussed previously (Storz et al. 2011, Mellin and Cossart 2012, Lybecker et al. 2014, Dutta and Srivastava 2018).

At 37°C, the predicted interacting region of *rplW* and *rplB*, which encode the ribosomal proteins L23 and L2, respectively, are in fact the same sequence due to the proximity of the two genes in the genome. In *B. subtilis, rplW* and *rplB* are arranged as part of an operon with other ribosomal proteins with *rplW* immediately upstream of *rplB* (Li et al. 1997); therefore it is possible that Rli47 binding at this position could abort translation of the other genes in the operon after *rplW* translation. L23 is part of the polypeptide exit channel, where it is implicated in the binding of the signal recognition particle and the trigger factor chaperone (Kramer et al. 2002, Nikolay et al. 2015), and L2 is an essential component of the peptidyl transferase center (Nakagawa et al. 1999). Nth is a DNA glycosylase involved in DNA repair with implications in virulence. An *nth* deletion mutant was more susceptible to H_2_O_2_, was less capable of colonizing the liver and spleen in a mouse model, and showed lower phagosomal survival (Zhang et al. 2020). LtrA is involved in growth at low temperatures (Zheng and Kathariou 1995), and an interaction with *ltrA* and Rli47 could therefore explain the temperature-dependent phenotype observed in this study. Additional research will be necessary to determine the interaction, if any, of Rli47 with these transcripts and the consequent effects on *L. monocytogenes* physiology and virulence.

Notably, *tsaE* and *menA* are implicated to affect the *L. monocytogenes* cell wall and cell membrane, respectively. TsaE is involved in the generation of N6-threonylcarbamoyl adenosine, an important modified nucleoside essential for the structure of ANN-decoding tRNAs (Nguyen et al. 2017). Knockout mutants of the *tsaE* homolog *ydiB* (also known as *ubk*) in *B. subtilis* and *Streptococcus pneumoniae* result in aberrations in cell morphology potentially due to the role of the gene in translation and/or uncharacterized protein kinase activity (Lauhon 2012, Nguyen et al. 2017, Luthra et al. 2018, Pelletier et al. 2019, El-Khoury et al. 2020). MenA is part of the menaquinone synthesis pathway where it prenylates 1,4-dihydroxy-2-napthoyl-coenzyme A into demethylmenaquinone prior to the final conversion to menaquinone (Smith Hans et al. 2021). In addition to its required role in the electron transport chain (Smith Hans et al. 2021), the presence of menaquinone in the *L. monocytogenes* membrane is required for membrane integrity at lower temperatures and in response to freeze–thaw cycles (Flegler et al. 2021). Interactions of Rli47 with *tsaE* and *menA* could therefore affect cell envelope composition, potentially resulting in a cell envelope structure that is protective against lactic acid stress; this could at least partially explain the differences in lactic acid survival of 6179 and 6179Δ*rli47* (Cotter and Hill 2003, Lund et al. 2014, 2020, Ramstedt et al. 2014, Manzo et al. 2021). While experimental validation will be necessary to confirm any interaction between Rli47 and any of the aforementioned transcripts, it is nonetheless interesting that the predicted interaction of Rli47 with *tsaE* involves the third stem loop of Rli47 and the *tsaE* Shine–Dalgarno region as this is highly reminiscent of the canonical Rli47-*ilvA* interaction.

Notably, *ilvA* was not predicted by CopraRNA as a target for Rli47 despite the presence of the *ilvA* gene in all seven *Listeria* strains investigated. Upon analysing the IntaRNA outputs for each organism at each temperature, we observed that the canonical interaction between the third stem loop of Rli47 and the *ilvA* transcript Shine–Dalgarno region was in some cases not one of the two most energetically favorable interactions between Rli47 and the *ilvA* transcript (Supplementary Files 9 and 10). Indeed, the canonical interaction was not predicted at 20°C for *L. monocytogenes* strains EGD-e and 10403S or *L. ivanovii*. This may suggest that Rli47 function, at least in terms of isoleucine synthesis, may be both temperature- and species/strain-dependent. Comparative analysis of the *rli47* and *ilvA* loci revealed that the GAGG motif of the *ilvA* Shine–Dalgarno sequence and CTCC motif of the *rli47* third stem loop were completely conserved across the seven strains analyzed (Supplementary Files 9 and 10). These two motifs have been experimentally verified to be critical for the interaction between Rli47 and the *ilvA* transcript. However, other sections of the predicted third stem loop predicted to interact with the *ilvA* transcript in EGD-e by (Marinho et al. 2019) did vary across the strains analyzed (Supplementary File 10). Additionally, variations in the rest of the Rli47 and *ilvA* sequences could alter secondary and tertiary structures of these RNAs, resulting in different binding affinities (Supplementary File 9). This variation in Rli47 and *ilvA* sequences may in part explain some of the discrepancies between our results and those of (Marinho et al. 2019).

### General discussion

Currently, the sole known function of Rli47 is disruption of isoleucine biosynthesis by binding to the *ilvA* transcript in the absence of isoleucine. The experiments conducted in this work suggest that Rli47 is involved in other areas of *L. monocytogenes* physiology, including under conditions where isoleucine is not limited. This possibility is particularly relevant for the growth of *L. monocytogenes* in dairy products where oligopeptides of casein (which contain high levels of isoleucine) and free isoleucine are highly prevalent (Liu and Ream 2008, Phelan et al. 2009, Hogenboom et al. 2017, Nielsen et al. 2022). Similarly, fermented meat products also possess high levels of free isoleucine (Hierro et al. 1999). Future research will be necessary to determine what role, if any, Rli47 plays in isoleucine-replete conditions, where it is unlikely to be mediating cell growth by its known function of interacting with the *ilvA* transcript.

Previous research has identified *rli47* homologs in species belonging to the *Listeria sensu stricto* clade, which have all been identified in mammalian gastrointestinal tracts or feces. However, *rli47* is notably absent from the environmentally associated *Listeria sensu lato* species (Marinho et al. 2019). Our BLASTn analysis further revealed that each of the more than 500 *L. monocytogenes* strains analyzed possessed a homolog of *rli47*. These homologs were highly conserved across all strains, with all *rli47* homologs being very similar to the EGD-e *rli47* sequence in terms of both length and % nucleotide identity. The conservation of *rli47* in mammalian-associated *Listeria* species and its absence from the *sensu lato* clade could suggest a potential role for *rli47* in the host environment.

Under the conditions investigated, we observed that logarithmic phase cultures of 6179Δ*rli47* exhibited higher rates of lactic acid survival than the wild type. While this strongly suggests that Rli47 is involved in the response of 6179 to lactic acid, the exact mechanism remains unclear. Still, the upregulation of *lmo2143* in 6179Δ*rli47* following lactic acid stress and potential interaction of Rli47 with *tsaE* and *menA* suggest that Rli47 could affect cell envelope structure, perhaps resulting in a cell envelope state more conducive to organic acid stress resistance. Lipidomics and other assessments of the cell membrane and cell wall could be used to determine any differences between 6179 and 6179Δ*rli47*. Additionally, the existing flow cytometry protocol could be modified to test the hypothesis that absence of *rli47* imparts higher lactic acid survival by means of increasing recovery of sublethally injured cells or increasing the population of persister cells. Future work will also be necessary to confirm the putative interactions between Rli47 and various mRNA transcripts suggested in the present *in silico* analysis.

Further characterization of the role of Rli47 in the stress response could be conducted by repeating the analyses across a range of pH. The cell survival phenotype of 6179Δ*rli47* would be expected to be less pronounced as pH increases because lactic acid would be less able to diffuse into the cell (Hirshfield et al. 2003). While the work by Mujahid et al. (2013) indicates that Rli47 is not involved in the strong acid response, future research would be necessary to determine if the *rli47* deletion phenotype is also observed in response to other organic acids, such as acetic acid, that have been shown to kill *L. monocytogenes* (Heavin et al. 2009, Arvaniti et al. 2021). The hypothesized mechanisms by which Rli47 is involved in lactic acid survival (e.g. cell envelope modulation) would likely be broadly protective against other organic acids, but additional experimentation would be necessary to determine if this is actually the case. Still, although the findings presented here do not elucidate the exact role of Rli47 in the *L. monocytogenes* stress response, this study provides further insight into the complex regulatory role of sRNAs in bacterial gene regulation.

## Conclusion

This study provides evidence that *rli47* plays a role in the *L. monocytogenes* stress response. The *rli47* deletion mutant 6179Δ*rli47* demonstrated a higher survival following lactic acid stress than its parent strain. Additional flow cytometry and RNAseq experiments further indicated that Rli47 function may vary depending on growth phase and nutrient availability. Transcriptomics and *in silico* predictions also suggested that Rli47 may be connected to cell envelope integrity. In conclusion, this study identifies a potential role for Rli47 in the saprophytic lifestyle of *L. monocytogenes* and suggests possible additional regulatory roles of this sRNA.

## Supplementary Material

xtaf012_Supplemental_Files

## Data Availability

The raw transcriptome sequencing data for this work as well as the long read data for 6179Δ*rli47* were submitted to the SRA under BioProject accession PRJNA1164155. Code associated with this work can be found at https://github.com/benc347/rli47_analysis.
