# Supplementary material for: Investigating the role of the Listeria monocytogenes noncoding RNA Rli47 during the response to environmental stressors
Source: FEMS Microbes. 2025 Oct 20;6:xtaf012. doi: 10.1093/femsmc/xtaf012 (PMC12596718; doi:10.1093/femsmc/xtaf012)
Supplement: xtaf012_Supplemental_Files [file xtaf012_supplemental_files.zip › FEMSMC-2024-052.R1 one sentence summary.docx]

The non-coding RNA Rli47 is involved in the Listeria monocytogenes stress response
