# Supplementary material for: Investigating the role of the Listeria monocytogenes noncoding RNA Rli47 during the response to environmental stressors
Source: FEMS Microbes. 2025 Oct 20;6:xtaf012. doi: 10.1093/femsmc/xtaf012 (PMC12596718; doi:10.1093/femsmc/xtaf012)
Supplement: xtaf012_Supplemental_Files [file xtaf012_supplemental_files.zip › Supplementary Tables 1 - 4 FEMS.docx]

**Supplementary Table 1: Primers used in this study.** Restriction sites are bolded, and the restriction enzyme is listed in parentheses. Underlined sections of Rli47B2 and Rli47C2 are complementary, allowing for annealing in SOE PCR.

| **Primer** | **Function** | **Sequence** |
| --- | --- | --- |
| RA | Forward primer amplifying region upstream of *rli47* | 5’ AAAA**CCATGG**TTATACATTTTACTCGCAGC 3’ (NcoI) |
| RB3 | Reverse primer amplifying the region upstream of *rli47* | 5’ CGATTTCGTCACGCGGGGTTTTTCACTATATATATAACC 3’ |
| RC2 | Forward primer amplifying the region downstream of *rli47* | 5’ GAAAAACCCCGCGTGACGAAATCG 3’ |
| RD | Reverse primer amplifying the region downstream of *rli47* | 5’ AACCAA**GTCGAC**GTTCATTGTTCTTCTTTTGGATGG 3’ (SalI) |
| pHOSS1_fwd | Forward primer amplifying part of pHOSS1 backbone | 5' TGAAGTTACCATCACGGAAAAAGG 3' |
| pHOSS1_rev | Reverse primer amplifying part of pHOSS1 backbone | 5' GATCTAATGATTCAAACCCTTGTG 3' |
| pHOSS1MCSF | Forward primer amplifying the pHOSS1 multiple cloning site | 5' CCTGGAGCTGGTATATAAGTCCC 3' |
| pHOSS1MCSR | Reverse primer amplifying the pHOSS1 multiple cloning site | 5' GGAAGCGAGAAGAATCATAATGGGG 3' |
| Rli47SeqF | Forward primer amplifying the *rli47* locus | 5’ GGCTACATTAAAATCGCCTTACG 3’ |
| Rli47SeqR | Reverse primer amplifying the *rli47* locus | 5’ CCTTGGAGGAATTAGTAACGC 3’ |
| Rli47ChromF | Forward primer amplifying ~1.2 Kb upstream and downstream of the *rli47* locus. Does not amplify the deletion allele cloned into pHOSS1 | 5' GCATCATGGAAGTCATTTTATCCAGCG 3' |
| Rli47ChromR | Reverse primer amplifying ~1.2 Kb upstream and downstream of the *rli47* locus. Does not amplify the deletion allele cloned into pHOSS1 | 5' CGTTATTTAATGCCCACTCATCCG 3' |
| ClpLF | Forward primer amplifying the 6179 plasmid gene *clpL* | 5’ TCATGTCGATCAAATCGATAGC 3’ |
| ClpLR | Reverse primer amplifying the 6179 plasmid gene *clpL* | 5’ AAGCAATTGTGGCTGGTAAAGT 3’ |
| Rli47CompF | Forward primer amplifying *rli47* for ligation into pNZ44 | 5’ CCC**TCTAGA**CAACGAACAAATAAAAGG 3’ (NcoI) |
| Rli47CompR | Reverse primer amplifying *rli47* for ligation into pNZ44 | 5’ CAC**AAGCTT**CAAAAAACCCCGAATGACG 3’ (HindIII) |
| pNZ44F | Forward primer amplifying the pNZ44 MCS | 5’ CTAATGTCACTAACCTGCCCCG 3’ |
| pNZ44R | Reverse primer amplifying the pNZ44 MCS | 5’ GCTTTATCAACTGCTGCT 3’ |
| Lip1 | Forward primer amplifying *prfA* | 5’ GATACAGAAACATCGGTTGGC 3’ |
| Lip2 | Reverse primer amplifying *prfA* | 5’ GTGTAATCTTGATGCCATCAGG 3’ |

**Supplementary Table 2: Annealing temperatures for primer pairs.** Primer pairs and associated annealing temperature. “NA” indicates that the primer pair was not used with the given polymerase.

| **Primer Pair** | **Annealing Temperature (°C) – Phusion DNA Polymerase** | **Annealing Temperature (°C) – Platinum II Taq DNA Polymerase** |
| --- | --- | --- |
| RA/RB3 | 67.3 | NA |
| RC2/RD | 69.5 | NA |
| pHOSS1_fwd/rev | NA | 56.6 |
| pHOSS1MCSF/R | NA | 64.6 |
| Rli47SeqF/R | NA | 58.1 |
| Rli47ChromF/R | NA | 60.7 |
| ClpLF/R | NA | 57.1 |
| Rli47CompF/R | 64.8 | 60.5 |
| pNZ44F/R | NA | 60.0 |
| Lip1/2 | NA | 64.0 |

**Supplementary Table 3: PCR components and volumes per reaction.** Reagents and volumes used for PCR reactions using Phusion High-Fidelity DNA Polymerase and Platinum II Taq Hot-Start DNA Polymerase.

| **Component for Phusion PCR** | **Volume (μL)** | **Component for Platinum II Taq PCR** | **Volume (μL)** |
| --- | --- | --- | --- |
| Phusion Mastermix (0.04 Units/μL Phusion DNA Polymerase, 2X buffer, 400 μM of each dNTP) | 25 | H_2_O | 13.64 |
|  |  | PlatTaqII buffer | 4 |
|  |  | 10 mM dNTPs | 0.4 |
| H_2_O | 19 | Forward primer | 0.4 |
| Forward primer | 2.5 | Reverse primer | 0.4 |
| Reverse primer | 2.5 | PlatTaqII polymerase | 0.16 |
| Template DNA | 1 | Template DNA | 1 |

**Supplementary Table 4: PCR cycling conditions for Phusion and Platinum Taq II Hot-Start DNA Polymerase.** Steps 2-4 were repeated for a total of 35 cycles.

|  | **Step** | **Temperature (°C) - Phusion** | **Time - Phusion** | **Temperature (°C) – Platinum II Taq** | **Time – Platinum II Taq** |
| --- | --- | --- | --- | --- | --- |
| 1 | Initial Denaturation | 94 | 2 minutes | 98 | 30 seconds |
| 2 | Denaturation | 94 | 15 seconds | 98 | 10 seconds |
| 3 | Annealing | (See Table2) | 15 seconds | (See Table2) | 30 seconds |
| 4 | Extension | 72 | 15 seconds per Kb | 72 | 30 seconds per Kb |
| 5 | Final Extension | 72 | 5 minutes | 72 | 5 minutes |
