# Supplementary material for: Investigating the role of the Listeria monocytogenes noncoding RNA Rli47 during the response to environmental stressors
Source: FEMS Microbes. 2025 Oct 20;6:xtaf012. doi: 10.1093/femsmc/xtaf012 (PMC12596718; doi:10.1093/femsmc/xtaf012)
Supplement: xtaf012_Supplemental_Files [file xtaf012_supplemental_files.zip › FEMSMC-2024-052.R1 supplementary data - reviews and author response.docx]

**Reviews of manuscript FEMSMC-2024-052**

**Reviewer 1**

**Recommendation: Major Revision**

Summary:

Here, the authors investigate Rli47, a ncRNA, in the Listeria monocytogenes 6179 background known to be FPE-relevant due to its persistence. Deletion of rli47 increased survival of logarithmic phase bacteria to 1% lactic acid, but did not affect the proportion of metabolically active cells nor gene transcription under various conditions. Differential gene transcription was observed in stationary cells stressed cells. In silico analysis predicts that Rli47 might bind to cell envelope-related factors that will be validated in the future.

Major:

- Fig. 1A: Are the labels swapped? As they are right now, I see that the mutant strain grows faster than the wild-type strain, independently of temperature. The text mentions though that there are no differences between the strains.

- Deletion of rli47 moderately increases survival at 2h, but have you performed a time-course to characterize the defect over time? By including a 30 min time point, it can inform about the killing kinetics at the time of harvest for RNA-Seq. Likewise, at longer time-points it could inform about the presence of more resistant sub-populations.

- One possibility for the discrepancies between other studies and this one is that the ∆rli47 acquired might have acquired a suppressor mutation that masks the phenotype. Have you performed WGS to rule it out?

- L506: CopraRNA did not predict ilvA as a target, even though it’s a known target of Rli47 in Listeria monocytogenes. It’s interesting that this mechanism might be strain specific. Have you confirmed that the ∆rli47 strain in your background does not have a shorter lag-phase in isoleucine-depleted media, aligning with your in silico analysis?

Minor:

- Since the stresses you selected are lactic acid, temperature, and H2O2, the background section would benefit of a brief description of when Listeria encounters these stresses in FPEs.

- Since you observe an increase in metabolically active cells upon lactic acid addition to stationary phase cells, does Listeria use lactic acid as a nutrient source?

- Paragraph starting at L480 would benefit of adding the possible function of the genes to help the reader.

**Reviewer 2**

**Recommendation: Major Revision**

In this manuscript the authors explore the impact of deleting the small RNA Rli47 from L. monocytogenes on growth and acid tolerance. Rli47 has been previously shown to play a role in the growth suppression of L. mono in the absence of isoleucine. Here they show that there is minimal impact of deleting Rli47 at different growth temperatures in TSB, but show that the small RNA is important for tolerance to lactic acid stress. Despite this observation, they indicate that there is no difference in the numbers of metabolically active cells, with and without the small RNA, when challenged with lactic acid. I have the following comments for the authors to address moving forward.

1. The authors should expand on the importance of acid tolerance in L. mono and specifically lactic acid. What is the overall advantage of encoding this small RNA and its role for this organism as a pathogen? There seems to be a lot of connections of the RNA to important phenotypes, but placing this into the importance of pathogenesis or physiology is lacking.

2. How common is this small RNA among strains of L. mono? A more in-depth analysis using comparative genomics would strengthen the importance of this work.

3. It would be helpful to extend this more broadly for acid stress. They use a weak acid (lactic acid), but do not provide much depth beyond this. First, does L. mono show a similar phenotype to other weak acids? What about strong acids like HCl? Is it all about protons and maintaining cell wall homeostasis or the influx of undissociated acid molecules?

4. To the latter point above. Since there is a link between the small RNA and isoleucine is there a connection to buffering capacity and amino acid production that is connected to lactic acid stress?

5. Finally, what about varying this across a range of pH. Does the phenotype respond to changes in pH beyond physiological pH?

**Associate Editor Recommendation: Major Revision**

**EIC Decision: Major Revision**

**Author's Response to Decision Letter for (FEMSMC-2024-052)**

Thank you for handling this matter and for finding reviewers for the manuscript. We have addressed the reviewer’s comments textually and performed as much additional experimentation as was possible. However, we were unable to perform extensive additional experiments due to major changes in our situations in the past year since we submitted the study (Senior author Stephan Schmitz-Esser has begun a new position at South Dakota State University, Main author Bienvenido Tibbs-Cortes was cut by the federal layoffs and no longer has laboratory access, and co-author Jessica Strathman has joined a new research lab at Iowa State University due to Dr. Schmitz-Esser departure from Iowa State University).

We agree that additional experimentation would of course improve this study, but we believe that it is still a valid contribution to the field. However, we would like to highlight the extensive nature of the additional experiments that the reviewers requested. While those experiments are surely scientifically justified, all those changes would require extensive lab work. For example, performing the same experiments in isoleucine-depleted chemically defined media or testing different pH ranges is, unfortunately, not feasible for us given the lack of personnel to be able to work on those experiments. We have acknowledged in the manuscript in multiple areas the importance of the additional experiments suggested by the reviewers. We were able to perform the whole genome sequencing of the rli47 deletion mutant suggested by Reviewer 1, and we hope that this confirmation of our mutant and as well as our responses to you and the reviewers are sufficient. Thank you again for your help.

Reviewers' Comments to Authors:

Reviewer: 1

Comments to the Author

Summary:

Here, the authors investigate Rli47, a ncRNA, in the Listeria monocytogenes 6179 background known to be FPE-relevant due to its persistence. Deletion of rli47 increased survival of logarithmic phase bacteria to 1% lactic acid, but did not affect the proportion of metabolically active cells nor gene transcription under various conditions. Differential gene transcription was observed in stationary cells stressed cells. In silico analysis predicts that Rli47 might bind to cell envelope-related factors that will be validated in the future.

Major:

- Fig. 1A: Are the labels swapped? As they are right now, I see that the mutant strain grows faster than the wild-type strain, independently of temperature. The text mentions though that there are no differences between the strains.

Response: Thank you for noticing the error. The figure labels were indeed swapped. We have updated the labels for figure 1A in the resubmission.

- Deletion of rli47 moderately increases survival at 2h, but have you performed a time-course to characterize the defect over time? By including a 30 min time point, it can inform about the killing kinetics at the time of harvest for RNA-Seq. Likewise, at longer time-points it could inform about the presence of more resistant sub-populations.

Response: We appreciate the input and agree that additional timepoints would be helpful in further assessing the effects of rli47 deletion on L. monocytogenes lactic acid survival. We originally chose the 2-hour time point as this duration of lactic acid exposure was previously tested in our lab (Naditz et al. 2019).

While we again agree that a full time-course would be useful, this is beyond the scope of what we can achieve given the changes with respect to personnel and resources available for this project. We believe that the existing results are still valid and informative. To accommodate the reviewer’s comment, we have addressed this point in lines 621-623 of the revised manuscript to state the importance of such experiments in future research.

- One possibility for the discrepancies between other studies and this one is that the ∆rli47 acquired might have acquired a suppressor mutation that masks the phenotype. Have you performed WGS to rule it out?

Response: Thank you for your input. We have performed WGS of the rli47 deletion mutant and subsequent variant calling. The methods and related discussion for the sequencing and variant calling are located at lines 223-256.

Briefly, we identified only a single SNP with a VCF quality score of >20. This SNP was found in lmo0459 resulting in a Ser to Leu mutation at residue 63 in Lmo0459, an uncharacterized gene. This mutation falls outside of the predicted helix-turn-helix domain in Lmo0459. Additionally, we performed 3D protein structure alignment comparing AlphaFold3 structures between Lmo0459 with and without the Ser to Leu mutation, and all metrics demonstrated that this mutation had little to no effect on the Lmo0459 protein structure.

Based on our analysis, we do not believe that these additional differences are likely to explain the observed phenotype, but we have clearly stated that confirming this would require additional research.

- L506: CopraRNA did not predict ilvA as a target, even though it’s a known target of Rli47 in Listeria monocytogenes. It’s interesting that this mechanism might be strain specific. Have you confirmed that the ∆rli47 strain in your background does not have a shorter lag-phase in isoleucine-depleted media, aligning with your in silico analysis?

Response: We appreciate the comment. Because we were investigating the upregulation of rli47 in response to lactic acid observed in our previous publication (Cortes et al. 2020), we utilized TSB in the present work to keep the growth medium consistent with our previous research. We therefore do not have data for 6179 and 6179Δrli47 growth kinetics in isoleucine-depleted media or any data relating to how stressors affect these two strains when grown without isoleucine. We agree with the reviewer that this is a limitation of our study. While we did not have the resources to complete additional testing in isoleucine-depleted media, we have further emphasized this as a limitation to this work in lines 590-593 of the revised manuscript.

Minor:

- Since the stresses you selected are lactic acid, temperature, and H2O2, the background section would benefit of a brief description of when Listeria encounters these stresses in FPEs.

Response: As suggested, we added more details on where L. monocytogenes encounters such stress conditions in FPEs (L 102-107) in the revised manuscript).

- Since you observe an increase in metabolically active cells upon lactic acid addition to stationary phase cells, does Listeria use lactic acid as a nutrient source?

Response: There is only limited data available regarding the use of lactate as a nutrient source by L. monocytogenes. One publication (Grubmuller et al., 2014, Front Cell Infect Microbiol. 2014;4:156. doi: 10.3389/fcimb.2014.00156 ) analyzed the carbon substrates used by L. monocytogenes during growth in macrophages. This study revealed that lactate may serve as a nutrient source for L. monocytogenes, but lactate is not an efficient growth substrate. Thus, based on this, we assume that lactate is not a relevant carbon source for L. monocytogenes and may only be partially contribute to the increase of metabolically active cells observed here.

- Paragraph starting at L480 would benefit of adding the possible function of the genes to help the reader.

Response: We agree with the reviewer and have added brief descriptions of the proteins encoded by these genes in the paragraph starting at L526 (previously starting at L480). We have left the more detailed descriptions of these genes and their potential roles in the discussion section (listed below).

lmo2360 and lmo2361 – described beginning around L695

lmo2114 and lmo2115 – described beginning at L707

lmo2143 - described beginning at L41

Reviewer: 2

Comments to the Author

In this manuscript the authors explore the impact of deleting the small RNA Rli47 from L. monocytogenes on growth and acid tolerance. Rli47 has been previously shown to play a role in the growth suppression of L. mono in the absence of isoleucine. Here they show that there is minimal impact of deleting Rli47 at different growth temperatures in TSB, but show that the small RNA is important for tolerance to lactic acid stress. Despite this observation, they indicate that there is no difference in the numbers of metabolically active cells, with and without the small RNA, when challenged with lactic acid. I have the following comments for the authors to address moving forward.

1. The authors should expand on the importance of acid tolerance in L. mono and specifically lactic acid. What is the overall advantage of encoding this small RNA and its role for this organism as a pathogen? There seems to be a lot of connections of the RNA to important phenotypes, but placing this into the importance of pathogenesis or physiology is lacking.

Response: Thanks for your comment. Indeed, acid stress as analyzed in this study and reported in other studies that analyzed L. monocytogenes’ gene expression is mostly relevant to conditions encountered by. L. monocytogenes in FPEs (Please also see our response to a comment by reviewer #1 above and L102-107). With regard to L. monocytogenes’ role as a human and animal pathogen, L. monocytogenes will encounter acid stress conditions in the gastrointestinal tract (particularly in the stomach) as well as during replication in macrophages.

2. How common is this small RNA among strains of L. mono? A more in-depth analysis using comparative genomics would strengthen the importance of this work.

Response: Thank you for the suggestion. We agree that information about the prevalence of rli47 across L. monocytogenes strains would be beneficial to the manuscript. We used BLASTn to search for homologs of rli47 across all 506 strains of L. monocytogenes with complete genomes in the NCBI RefSeq database. Homologs were found in all strains with a minimum percent nucleotide identify of 96.47%. We have updated the methods (lines 422-427), results (lines 540-543), and discussion (lines 830-838) to incorporate the comparative genomics. The raw BLASTn table has also been added as supplemental file 8.

3. It would be helpful to extend this more broadly for acid stress. They use a weak acid (lactic acid), but do not provide much depth beyond this. First, does L. mono show a similar phenotype to other weak acids? What about strong acids like HCl? Is it all about protons and maintaining cell wall homeostasis or the influx of undissociated acid molecules?

Response: In general, L. monocytogenes responds similarly to other weak organic acids. As the reviewer points out, for organic acids such as lactic acid, the equilibrium between the dissociated und undissociated lactic acid molecules is a key point. In general, L. monocytogenes is able to prevent free protons from entering the cells, but the diffusion of undissociated weak acids such as lactate into the cytoplasm is the main effect of lactic acid at pH values below its pKa (3.8) as in our work. We have added a section acknowledging that further analysis is necessary to determine how Rli47 is involved in the response to other organic acids at lines 855-861.

4. To the latter point above. Since there is a link between the small RNA and isoleucine is there a connection to buffering capacity and amino acid production that is connected to lactic acid stress?

Response: To the best of our knowledge, neither isoleucine itself nor isoleucine production are involved in buffering capacity. Based on the data on rli47 from this study and our previous study (Cortes et al., 2020), we cannot make statements about the potential role of isoleucine as all experiments were performed in complex, undefined rich media (TSB). Please also see our response regarding the importance of future research to investigate the role of isoleucine in more detail using chemically defined media Lines 590-593.

5. Finally, what about varying this across a range of pH. Does the phenotype respond to changes in pH beyond physiological pH?

Response: Thank you for your comment. The pH of the media used in this study (pH ~3.4) is well below the physiological pH of L. monocytogenes. However, we did not test additional pH levels, and therefore we do not know how the rli47 deletion phenotype responds across a range of pH. We agree that this would be important and relevant information. To accommodate the reviewer’s comment, we have included a sentence in the revised manuscript (L852-855) mentioning that studying the phenotype in response to different pH values would be relevant for future research

**Reviews of manuscript FEMSMC-2024-052.R1**

**EIC Decision: Accept**
